# Uncovering hundreds of exogenous and endogenous RNA viral RdRp sequences amongst uncharacterized sequences in public protein databases

**DOI:** 10.1093/ve/veaf074

**Published:** 2025-09-18

**Authors:** Katherine Brown, Andrew Edwin Firth

**Affiliations:** Division of Virology, Department of Pathology, University of Cambridge, Cambridge, United Kingdom; Division of Virology, Department of Pathology, University of Cambridge, Cambridge, United Kingdom

**Keywords:** RNA virus, metatranscriptomics, virus discovery, paleovirology, RdRp, endogenous viral elements

## Abstract

Public databases of protein sequences, such as the National Center for Biotechnology Information (NCBI) Protein repository and UniProt, contain millions of proteins identified in samples from specific species but named as uncharacterized or hypothetical due to a lack of information about their function. Many such sequences are actually derived from RNA viruses, either due to viral infection of the original sample, contamination, or endogenous viral elements (EVEs) integrated into the genome of the sample species. Many proteins from RNA virus discovery research are also deposited into these repositories but are labelled as uncharacterized and only classified taxonomically at a superkingdom or realm level. Sequences from protein repositories not labelled specifically as being derived from the RNA-viral RNA-dependent RNA polymerase (RdRp) protein are often used as negative controls when looking to identify viral RdRp sequences, so the presence of unlabelled viruses amongst these datasets is problematic. These sequences also represent a source of information about novel viruses and EVEs. In this study, we screened uncharacterized proteins from two large public protein repositories—NCBI Protein and UniProt—to identify sequences likely to be derived from RNA viral RdRp and to perform detailed characterization of sequences of interest. We identified 3560 such sequences, many derived from EVEs. Many are previously unknown EVEs, which led to characterization of additional, related sequences. For example, a group of orbi-like viruses infecting nematodes was uncovered that appears to have both ancient endogenous and circulating exogenous members. Many integrations of mito-like viruses into plant genomes were also found. In several host taxonomic groups, the first example of an EVE, and in some cases the first example of any RNA virus, was uncovered. The large number of EVEs uncovered by this relatively small-scale search suggests that only a fraction of the true diversity of EVEs is currently known. We also provide provisional taxonomic annotations for RdRps, currently only listed as members of the *Riboviria* realm. A number of sequences are identified that are indistinguishable from viruses but are labelled as bacteria, seemingly as a result of mislabelling or contamination. Non-RdRp sequences that share near-significant similarity with RdRp are also characterized. Finally, recommendations are made for generating useful negative controls and sets of negative control sequences are provided.

## Introduction

Over recent years, there has been an explosion in large-scale screening of high-throughput RNA-sequencing (RNA-seq) datasets for evidence of RNA viruses (Shi et al. 2016, Webster et al. 2016, Zhang et al. 2018, Gilbert et al. 2019, Rosani et al. 2019, Callanan et al. 2020, Charon et al. 2020, Wolf et al. 2020, Wu et al. 2020, Chen et al. 2022, Edgar et al. 2022, Forgia et al. 2022, Mifsud et al. 2022, Neri et al. 2022, Zayed et al. 2022, Hou *et al.* 2024, Olendraite et al. 2023). Most studies focus on identification *via* similarity to the RNA-viral RNA-dependent RNA polymerase (RdRp) gene, the only gene that is conserved across all RNA viruses. Several tools have been published to aid in RNA virus detection in assembled RNA-seq data, for example, RdRp-scan (Charon et al. 2022), PalmScan (Babaian and Edgar 2022) VirSorter and VirSorter2 (Roux et al. 2015, Guo et al. 2021), and LucaProt (Hou et al. 2024).

RNA virus discovery research requires robust negative control sets of diverse, biologically derived sequences, known not to be derived from viral RdRp, to eliminate or reduce false positives. Approaches based on machine learning also require a training set of known nonviral sequences. An intuitive option is to use one of the many publicly available protein databases as a source of such sequences, with sequences annotated as non-RNA viral in origin selected as negative controls. Putative viruses that show higher similarity to any sequence in this negative control set than to any RNA virus are discarded as false positives.

Many successful virus discovery projects have used this approach, using similarity against proteins not annotated as RNA viruses from the NCBI non-redundant protein (nr) database (Sayers et al. 2021) or the Pfam database (Mistry et al. 2021) to eliminate potential false positives (for example, Shi et al. 2016, Webster et al. 2016, Gilbert et al. 2019, Charon et al. 2020, Wu et al. 2020, Chang et al. 2021, Chen et al. 2022, and Zayed et al. 2022). However, often, these databases contain nucleotide or protein sequences that are derived from viral RdRp but are unannotated or misannotated. Also, many cellular genomes contain integrated endogenous viral elements (EVEs), derived from RNA virus sequences entering the genome of their host *via* chromosomal integration. These are genuine host proteins, but, given that they originate from RdRp, they do not represent false positives for RdRp detection software and therefore eliminating sequences similar to these proteins is overly stringent.

Often, these unlabelled viral RdRp sequences will not be named as specific non-RNA-viral proteins but instead have labels such as uncharacterized, unknown, putative or hypothetical protein, or ‘domain of unknown function’. These labels are used when sequences are determined, either by the researcher or automatically, not to meet the criteria (based on homology, prediction of function, or experimental evidence) to be assigned to a specific protein family. The sequences are often taxonomically assigned based on the organism that was the main source of the biological sample. However, biological samples ostensibly from a single organism will often also contain viral RNA from active infection or transcribed EVEs in the host, its environment, or another source (Mifsud et al. 2022).

These unlabelled proteins, when derived from RNA viruses, hold great interest not only as a source of noise in virus discovery research, but also simply as a source of information about the RNA viruses present in biological samples. Despite virus discovery projects such as those listed above, novel viruses are still frequently discovered and provide valuable insight into virus host range, transmission patterns, and evolution. There are still host species, and in some cases whole host taxa, about the virome of which we know little. Taxa with poorly understood viromes tend to correspond to those with poorly annotated proteins, as under-studied species will have less data available to characterize genes. Therefore, uncharacterized proteins are likely to be a rich source of information about novel viruses infecting these species. EVEs in particular are underexplored in comparison to exogenous viruses and are often dismissed as confounding factors in virus discovery research but are an essential key to our understanding of both the evolutionary history and the current distribution of RNA viruses. Therefore, the intention of this study is not only to identify RdRp sequences amongst uncharacterized proteins, but to perform detailed characterization of any identified sequences of interest.

In this study, we present the results of large-scale screening of uncharacterized proteins from the National Center for Biotechnology Information (NCBI) Protein (Sayers et al. 2021) and UniRef (Suzek et al. 2015) databases for RNA viral RdRp proteins, in order to clarify the origin of these proteins and facilitate RNA virus discovery research. We characterize hundreds of new RdRp-like proteins, predominantly derived from RNA viral EVEs. These results significantly enhance our understanding of the distribution of EVEs amongst under-studied species. We also provide taxonomic information for hundreds of known but incompletely labelled RNA viral RdRps, list a number of likely mislabelled or contaminant-derived sequences, characterize non-RdRp sequences that share near-significant similarity with RdRp, and provide two sets of pre-screened negative control proteins as a resource for method development and RNA virus discovery research.

## Results

Over 300 million sequences labelled as unknown, unclassified, or similar were identified for screening by searching the NCBI protein and UniRef100 databases—75 483 361 from UniRef and 233 325 610 from NCBI.

Profile HMMs (pHMMs) have been previously shown to be a sensitive and accurate technique for detecting viral proteins (Charon et al. 2022, Olendraite et al. 2023); therefore, a pHMM-based approach was used to initially detect candidate RdRps. Pre-existing profiles from two recent publications (Charon et al. 2022, Olendraite et al. 2023) and from the Pfam (Mistry et al. 2021) database were used to ensure a comprehensive search.

**Figure 1:**
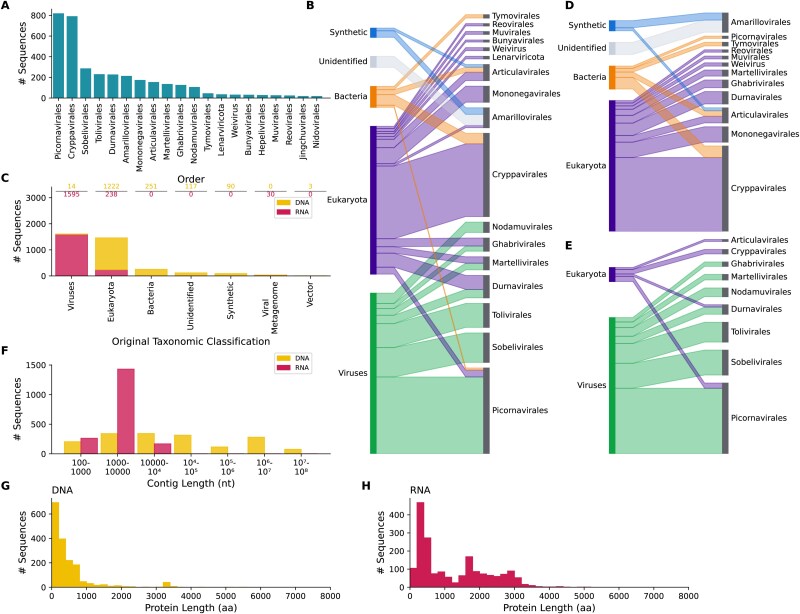
Summary of initial results. (a) Bar chart showing the number of RdRp-like uncharacterized proteins classified *via* phylogenetic analysis into each of the ICTV orders into which at least 10 proteins were classified. (b) Sankey plot comparing the classification of all identified RdRp-like uncharacterized proteins based on their NCBI taxonomy ID with the viral orders into which they were assigned. (c) Bar chart comparing the number of uncharacterized sequences with each classification based on their NCBI taxonomy ID for those labelled as having a DNA (yellow, top) or RNA (pink, bottom) source. Numbers above bars correspond to bar heights. (d, e) Sankey plots as in (b) but for DNA source sequences (d) or RNA source sequences (e) only. (f) Histogram showing the length distribution of source contigs categorized by their source nucleic acid type. (g, h) histogram indicating the protein sequence length distribution of uncharacterized proteins categorized by their source nucleic acid type.

An initial 92 741 uncharacterized sequences had significant matches (HMMER domain score ≥ 25) against these pHMMs. After additional filtering (described below), 3560 sequences were identified as likely to be RNA viral RdRp—1609 classified as Viruses/*Riboviria* (but not *Orthornavirae*) in their source database, 1460 as Eukaryota, 251 as Bacteria, 210 as synthetic, unidentified or vector, and 30 as metagenomic (Fig. 1).

### Filtering reverse transcriptase

Of the 92 741 sequences identified with the original set of RdRp pHMMs, a large majority (90%) matched one of two profiles: the Pfam profiles PF05919 (Mitovirus RNA-dependent RNA polymerase) and PF00680 (Viral RNA-dependent RNA polymerase). However, inspection of these sequences revealed that many have higher homology to the retroviral reverse transcriptase (RT) protein (and, correspondingly, retrotransposon RT) than to RNA viral RdRp. RT sequences are appropriate negative controls for RNA virus discovery projects, as RdRp and RT share distant homology (Peyambari et al. 2021), and RT is a common source of false positives.

In order to identify RT-like hits amongst the virus-like unclassified proteins, a phylogenetic analysis was performed on the sequences included in input alignments used to generate the two Pfam profiles, PF05919 and PF00680. (Fig. S1). In both cases, a monophyletic group—primarily of named RdRp proteins—formed, clearly distinct from RT sequences. Sequences with known RT domains, such as maturase and bacterial group II introns, clustered with RT.

New pHMMs were generated by dividing the Pfam profile input sequences into RdRp-like and RT-like clusters. All hits against PF05919 and PF00680 were compared to these pHMMs individually, and designated as RdRp or RT depending on the highest score. Removing sequences with higher similarity to RT than RdRp left 9249 putative viral sequences.

### Identifying RdRp

Several additional analyses were performed to determine if sequences were truly RNA viral in origin.

Sequences were assigned using phylogenetic analysis to one of 330 viral operational taxonomic units (OTUs). ESM Fold (Lin et al. 2023) predicted protein structures of all sequences were compared to Protein Data Bank (PDB) virus profiles using FoldSeek (van Kempen et al. 2024), PalmScan (Babaian and Edgar 2022) was used to identify core RdRp regions, and BLASTP (Altschul et al. 1990, Camacho et al. 2009) was used to make an additional sequence similarity comparison to known RdRp sequences. Sequences were required to score highly on at least two of the following criteria to be classified as RdRp-like: (i) a FoldSeek bit score of >100 against an RdRp profile, (ii) an identified RdRp core using PalmScan, (iii) a BLAST bit score of >50 against a known RdRp sequence, (iv) a branch length of <1.5 separating the sequence from the nearest known RNA virus in phylogenetic analysis, and (v) an HMMER domain score > 50 against an RdRp profile. After this filtering step, 3560 sequences remained. For each identified RdRp, Supplementary Table 1 lists the FoldSeek score and Pfam ID of the highest-scoring RdRp profile against the sequence, PalmScan score, BLAST bit score and highest-scoring match against an RNA virus, phylogenetic distance from and name of closest relative in phylogenetic analysis and HMMER domain score, and nearest matching HMMER profile.

The closest homologues to RdRp are RT and its relatives, to remove any sequences likely to be RT rather than RdRp, sequences with a high-scoring BLAST match against RT or with an RT-like core domain identified by PalmScan, were excluded.

An important caveat is that, as the NCBI protein database is not curated, many of the sequences identified here will not be unique but will be the result of either multiple projects sequencing the same genome, chromosome, or other sequence or overlapping contigs or scaffolds from the same study. Therefore, ‘sequences’ in this context refer to individual NCBI protein/UniProt records, rather than unique biological entities.

### RdRp diversity

The largest proportion of sequences identified here, 820 sequences, represent *Picornavirales*, a large order of positive-sense RNA viruses, followed by the *Cryppavirales* (792 sequences), another positive-sense order of RNA viruses containing only one family, the *Mitoviridae* (Walker et al. 2022) (Fig. 1a and b). The distribution of sequences between orders was very distinct depending on the assigned taxonomic origin of the original database entry (Fig. 1b). The majority of the *Picornavirales*, *Sobelivirales*, *Tolivirales*, and *Nodamuvirales* sequences were already classified as viral (but not RNA viral) in their source databases, while most *Cryppavirales*, *Mononegavirales*, *Articulavirales*, and *Bunyavirales* were classified as eukaryotic.

As RNA viruses only use RNA as their genetic material, it was expected that RdRp-like sequences would be largely derived from RNA, for example, sequences assembled from RNA-seq datasets. However, surprisingly, 48% of the RdRp-like proteins are labelled in their source database as originating from genomic DNA (Fig. 1c). Many protein database records are inferred open reading frames (ORFs) identified automatically *via* gene prediction on DNA sequences, using tools such as Augustus (Stanke et al. 2006). Therefore, the protein records link directly back to the DNA records without messenger RNA (mRNA) intermediates.

There are three possible sources of RNA viral material in DNA sequences—contamination, mislabelling, and EVEs. In the case of contamination or mislabelling, we would expect the DNA-source RdRps to resemble those with an RNA source. However, EVEs differ from circulating exogenous viruses in a number of ways.

Firstly, the host and virus taxonomic distribution of EVEs displays a number of biases that differ from those of exogenous viruses. The eukaryotic DNA putative RdRps (EDRdRps) examined here most often originate from plants (656/1222 sequences) and arthropods (274/1222 sequences). Arthropods are known to have EVEs, particularly from the *Mononegavirales*, *Martellivirales*, and *Durnavirales* (Gilbert and Belliardo 2022), while plants have many *Cryppavirales* and *Durnavirales* EVEs (Chu et al. 2014, Bruenn et al. 2015, Mifsud et al. 2022). Arthropod and plant EVEs are rarely derived from the *Picornavirales*, an abundant exogenous order amongst these hosts (Le Gall et al. 2008). The EDRdRps examined here are most commonly classified as *Cryppavirales* (52% of sequences), *Mononegavirales* (11%), and *Durnavirales* (9%) (Fig. 1d). Meanwhile, *Picornavirales* are rare, with only eight eukaryotic sequences identified. These percentages differ from the RNA-source RdRps, amongst which *Picornavirales* make up 42% of sequences, while *Cryppavirales*, *Mononegavirales*, and *Durnavirales* are 3%, 2%, and 6%, respectively (Fig. 1e). Therefore, the taxonomic distribution of the EDRdRps is more consistent with that of EVEs than of RNA viruses.

Secondly, EVEs will be integrated into host chromosomes, so the source nucleic acid sequences—chromosomes, scaffolds, or contigs—would be expected to often be of a chromosome-like length, i.e. on a megabase scale. Meanwhile, the longest known RNA viral genome is a 64 kb member of the *Nidovirales* (Neuman et al. 2025), so RNA viral transcripts are substantially smaller. Here, the DNA-source RdRps have a mean source sequence length of 14.7 Mb, compared to 5402 nt for the RNA-source RdRps sequences, an increase of >2500-fold (Fig. 1f). The majority of RNA source sequences are between 1 and 10 kb in length. This again suggests that the eukaryotic DNA-source sequences generally represent EVEs.

Thirdly, once EVEs enter the host genome, they are often no longer subject to selection pressure to maintain intact ORFs (Katzourakis et al. 2005), so the ORFs may be short and degraded compared to those of exogenous viruses. Therefore, the protein sequences of EVEs should be shorter, on average, than those of RNA viruses. Accordingly, the DNA-source RdRps here have a mean length of 482 amino acids, while the RNA-source RdRps have a mean length of 1228 amino acids (Fig. 1g and h).

Taken together, these observations suggest that EVEs are numerous amongst our DNA-source RdRp sequences.

### EVEs derived from positive-strand RNA virus RdRps

#### Cryppavirales

A high proportion (52%) of the identified EDRdRps cluster within the *Cryppavirales* order, which contains one family, the *Mitoviridae* (Fig. 1d) (Walker et al. 2022). Almost all of these sequences (637/640) are from plants from the Streptophyta clade and 636/640 from flowering plants of the Magnolopsida clade.

**Figure 2:**
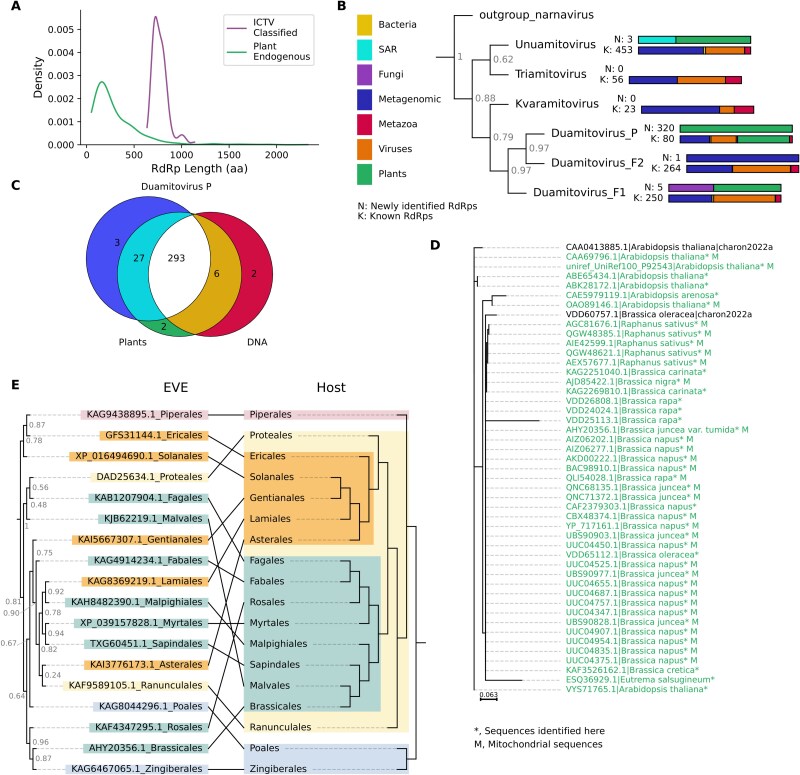
Mito-like EVEs. (a) Density plot showing the length distribution of plant DNA source uncharacterized proteins classed as mito-like in comparison to proteins from ICTV classified *Mitoviridae* species. (b) Summarized phylogenetic tree showing the distribution of the *Mitoviridae* genera (full phylogeny is available as Fig. S2). The tree has been simplified to show only sequences clustering monophyletically with members of ICTV-ratified genera and exclude sequences that did not fall clearly into any of these genera. Bars at node tips show the number of sequences in each group, the top bar (N, new) is the number of newly identified sequences and the bottom bar (K, known) the number of previously annotated sequences. Bar colours show the taxonomic origin of the sequences. (c) Venn diagram showing the overlap in sequences assigned to the *Duamitovirus* P clade, sequences identified in plants, and sequences with a DNA source. (d) Expansion of a single clade from the mito-like virus phylogeny. This clade has branch support of 1. Sequences marked with asterisks and with green text are newly identified; sequences with the suffix _M are from the mitochondrial genome. (e) Tanglegram comparing the phylogenetic relationships of the most common clade of mito-like viruses identified in each taxon (left) (approximate maximum likelihood tree generated with FastTree2 (Price et al. 2010) with those of Magnoliopsida plants (right), generated manually based on (Yang et al. 2020). Colours correspond to plant taxa.

The plant DNA-source *Mitoviridae*-like RdRps identified here have a mean ORF length of 305 amino acids, compared to 774 amino acids for ICTV classified *Mitoviridae* (Fig. 2a). This suggests that these are either partial sequences or EVEs. Many plant genomes are known to contain *Mitoviridae*-like (mito-like) insertions, particularly in the mitochondrial genome (Marienfeld et al. 1997, Nibert et al. 2018, Roossinck 2019, Begeman et al. 2023). Mito-like EVEs in plants are thought to have originally integrated into plant mitochondrial DNA, but then, in some cases, have been transferred into the nuclear genome (Charon et al. 2021). Accordingly, approximately one-quarter (170/637) of our identified plant DNA-source mito-like viruses are from sequences labelled as mitochondrial.

An additional 48 plant and 4 nonplant RNA-source mito-like RdRps were also identified. Thirty-six of these RNA-source sequences are mRNA sequences of mitochondrial proteins (eukaryotic mRNA putative RdRps, EMRdRps, to denote sequences specifically tagged as mRNA and mapped to a position in the host genome, rather than transcripts assembled only from RNA-seq), so they most likely represent transcribed sequences from the integrated RdRps, rather than transcripts from exogenous viruses.

The basic structure of the phylogeny of these sequences, along with previously identified *Mitoviridae* and mito-like sequences, is shown in Fig. 2b. The full phylogenetic tree is shown in Fig. S2. The major taxonomic groups of *Mitoviridae* were apparent, with the ICTV-classified *Duamitovirus*, *Unuamitovirus,* and *Triatomitovirus* genera forming monophyletic clades, together with many unclassified viruses (Fig. 2b, Fig. S2).

Almost all of the RdRps (320/333) in our study fell into a single, large clade within the *Duamitovirus* genus, designated here as the *Duamitovirus* Plant (P) clade (Fig. 2c). All but 3 of these sequences were from plants, and 293 had a DNA source. This clade also included 80 sequences from other studies (Fig. 2b), including all of the eight ICTV classified Magnoliospida *Duamitovirus* sequences (Nibert et al. 2018), plus one classified *Duamitovirus*, from an aquatic fern, *Azolla filiculoides*.

Within the P clade, many clusters of RdRp-like sequences are apparent. One clade of interest is isolated in Fig. 2d, containing a number of novel EVEs in members of the *Brassicales* order. This clade includes a protein known to represent an *Arabidopsis* EVE (P92543, Nibert et al. 2018) and additional EVEs from a number of members of the Brassicaceae family of plants, from the genera *Brassica*, *Raphanus*, *Arabidopsis*, and *Eutrema* (Fig. 2d). Two sequences from Brassicaceae from the RdRp-scan database are also in this clade. Particularly between members of the *Brassica* and *Raphanus* genera, there is almost no variation between these sequences. The closest ICTV classified virus is Oxybasis rubra mitovirus 1 (*Duamitovirus oxru1*); however, the Brassicaceae EVEs form a distinct monophyletic group.

Additional mitochondrial sequences were screened for viruses highly related to those in this clade (TBLASTN identity > 90%, alignment length > 100, bit score > 100). Related sequences were identified in the mitochondrial genome of species of the genera *Arabidopsis*, *Arabis*, *Barbarea*, *Brassica*, *Cardamine*, *Crucihimalaya*, *Lepidium, Nasturtium*, and *Raphanus*, all of which are members of the Brassicaceae family. None of the remaining mitochondrial genomes, including 27 complete mitochondrial genomes, from non-Brassicaceae members of the Rosid group, had these EVEs.

Assuming that the ORF of Oxybasis rubra mitovirus 1 (DAB41745.1) represents the approximate full-length ORF at 899 amino acids, none of these endogenous ORFs in Brassicaceae mitochondria are full length. A 234 amino acid region, including motifs A, B, and C, is common to all of the species in which the EVE was identified and is highly conserved, with >98% amino acid identity between all pairs of species—a maximum of four amino acid differences.

Mitochondrial genomes from the species of Brassicaceae with this EVE were aligned to determine if the insertion site of the EVE is at the same position in the mitochondrion, which is indicative of a virus that integrated prior to the divergence of the host species from a common ancestor. Shared insertion sites were identified based on aligned regions > 8000 nt in length containing the EVE but also non-EVE flanking sequences. The insertion site was found to be shared between *Arabidopsis*, *Barbarea*, *Cardamine*, *Crucihimalaya*, *Lepidium*, and *Nasturtium* and between *Brassica* and *Raphanus* species (Fig. S3a and b, alignment coordinates provided in Supplementary Data). This first group is consistent with a major phylogenetic group of Brassicaceae, discussed as clade A in Guo et al. (2017) (Fig. S3c). The conservation of the insertion site with clade A gives this insertion a minimum integration date of ~20 million years ago (MYA) (Fig. S3c). By examining the ORFs in the regions upstream and downstream of the insertion site, it is apparent that the genomic context of the EVE is similar in all the clade A species, with the largest EVE ORF falling between ORFs representing NADH-quinone oxidoreductase subunit H and ATP synthase subunit alpha (Fig. S4).

*Brassica* and *Raphanus* fall within clade B of the Brassicaceae and share a common ancestor approximately five MYA (Guo et al. 2017). These species again have a common insertion site and genomic context for this EVE (Fig. S3b and c). The genus *Arabis* is also in this clade but is more distantly related, with a common ancestor with *Brassica* and *Raphanus circa* 19 MYA (Fig. S4, Guo et al. 2017). The genomic context of the EVE differs between *Arabis* and the other clade B genera, and the *Arabis* mitochondrion could not be reliably aligned with either *Brassica* or *Raphanus* mitochondria.

These results initially suggest that this virus entered the genome of the Brassicaceae at least three times, once in the common ancestor of clade A at least 20 MYA, once in the ancestor of *Brassica* and *Raphanus* at least 5 and no >19 MYA, and once in the ancestor of the *Arabis* genus, within the last 19 million years. However, it is very unlikely for an RNA virus to retain this level of sequence conservation while circulating exogenously. Therefore, it is more likely that these insertions are instead the result of a single integration event followed by recombination, which is frequent in plant mitochondrial genomes (Morley and Nielsen 2017), or retrotransposition and then loss of some copies of the EVE.

The tanglegram in Fig. 2e shows the relationship, at host taxonomic order level, between the host phylogeny (Yang et al. 2020) and the largest group of endogenous mito-like RdRps identified in each order. It is not possible to recapitulate the host phylogeny using the mito-like sequences. This suggests multiple independent integration events. There are some similarities between the trees, for example, the Malpighiales, Myrtales, and Sapindales, all of which are Rosids, cluster together, as do the Ericales and Solanales, which are both asterids. However, overall, the trees are not highly similar; therefore, it is likely that the insertion of these elements into the genomes of their hosts occurred after the divergence of the host orders from a common ancestor.

### EVEs derived from negative-strand RNA virus RdRps

#### Mononegavirales

Amongst the eukaryotic RdRps resembling those of negative-strand RNA viruses, those derived from members of the order *Mononegavirales* were the most abundant, with 161 sequences. Of these, 142 are EDRdRps, and 11 are EMRdRps. The remaining eight are from assembled RNA-seq datasets and therefore cannot be assumed to be endogenous. The *Mononegavirales* sequences are primarily from arthropods (96 sequences) and from a specific member of the Acanthocephala group of parasitic worms, *Pomphorhynchus laevis* (18 sequences).

Figure S5A shows the phylogeny of 15 of the 18 *P. laevis* sequences. A sequence from the Serratus screening project (Edgar et al. 2022), also from *P. laevis,* is within this group, and, as this project used RNA-seq data, these RdRps must at least sometimes be transcribed. No viruses infecting this host have otherwise been previously described. These sequences cluster close to the *Cytorhabdovirus* genus of *Rhabdoviridae*. Several viruses isolated from arthropods are ICTV-classified members of this group, including viruses from Crustacea, a group used as intermediate hosts by *P. laevis* (Mauer et al. 2020). All of the *P. laevis* RdRp-like sequences are from unplaced contigs from a single genome project (Mauer et al. 2020), the only assembly available for any member of the Acanthocephala, so it is not clear how widespread this putative EVE is within this group. It is also not possible to say definitively that these contigs are from the specified host, rather than another species present in the sample, especially given that the assembly is derived from a host (barbel fish *Barbus barbus*) intestinal sample. However, the sequences are relatively divergent (mean 60% identity), fragmented in different ways and contain multiple insertions and deletions, which suggests that there are multiple insertions in the host genome and that at least some of the EVEs are ancient.

Amongst the 96 arthropod EVEs identified, 60 are from species of Lepidoptera and are widely distributed through the *Mononegavirales* phylogeny, with clusters related to the *Nyamiviridae*, *Xinmoviridae*, *Rhabdoviridae,* and *Artoviridae*. Some of the *Rhabdoviridae*-like sequences are shown in Fig. S5b.

A small cluster of RdRp-like EVEs was identified in *Perkinsus olseni*, a parasitic single-celled protist that infects molluscs (Fig. S5c). Nine sequences were found across three genome assemblies for this species. These are the first EVEs that have been identified in the Perkinsozoa phylum, and no RNA viruses have been described to date infecting this phylum except a single fragment of a toti-like RdRp identified in *Perkinsus chesapeaki* (Charon et al. 2022). The *P. olseni* sequences we identified here are divergent from any known virus family. The closest related group is the *Lispiviridae* (Fig. S5c); however, the highest-scoring BLAST hit against any ICTV-classified virus shares only 23% identity (KAF4688156.1 against YP_010800584.1 from *Copasivirus ivindoense*, Isopteran arli-related virus OKIAV103). The most closely related RNA virus overall is Macrotermes bellicosus lispivirus 1, with 23.61% identity, identified previously in a termite species (Wu et al. 2024). The sequences are fragmented and from different parts of the RdRp gene; however, they can be combined into a 1272 amino acid consensus, as shown in Fig. S6. Across the aligned regions, excluding gaps, there is a minimum of 82% identity between sequences, and a mean of 91% identity. A HHpred (Söding et al. 2005) search confirms similarity along the majority of this consensus (976/1272 amino acids) to the ‘Mononegavirales RNA-dependent RNA polymerase’ (PF00946.23) and ‘Mononegavirales mRNA-capping region’ (PF14318.10) Pfam records. To confirm that these EVEs are likely to be encoded by *P. olseni*, rather than by its mollusc hosts, the contigs containing the EVEs were examined. The longest *P. olseni* contig that contains a *Mononegavirales*-like EVE is 103 570 nt in length (JABANP010000160.1) and encodes several proteins with orthologues in other members of the *Perkinsus* genus, so, assuming this assembly is correct, this EVE appears to be part of the *P. olseni* genome.

### EVEs derived from double-stranded RNA viruses

#### Durnavirales

**Figure 3:**
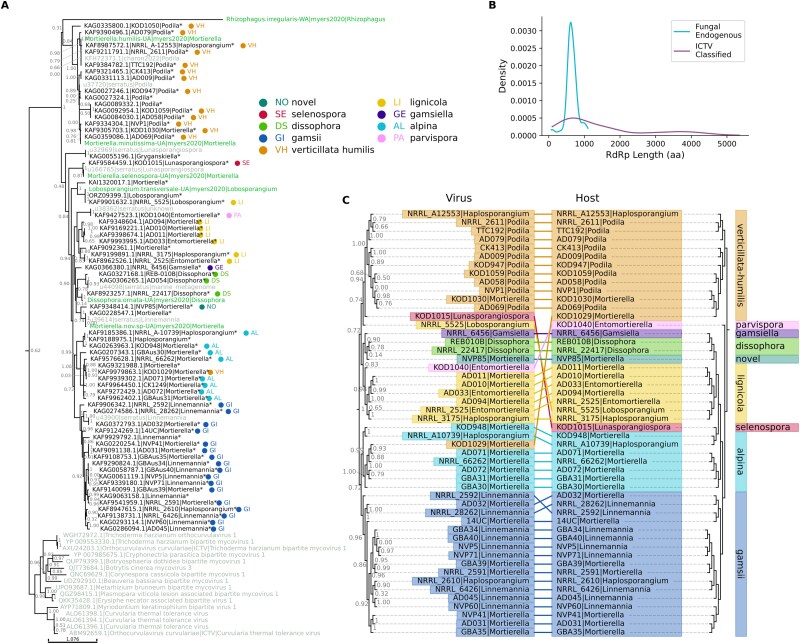
Durnavirales-like EVEs. (a) Approximate maximum likelihood (Price et al. 2010) phylogenetic tree showing the newly identified clade containing EVEs from the Mortierellaceae family of fungi and related sequences. The full phylogeny is available in the supplementary data. Node tips marked with a dot were sequenced as part of (Vandepol et al. 2020) and are labelled with the cluster into which the host species were classified by these authors (named hereafter as Vandepol clusters), as shown in the legend. These sequences are labelled with their GenBank accession, the ID originally assigned to the fungal isolate and their GenBank genus. Newly identified sequences are marked with an asterisk (*). Sequences with the label myers2020 and shown in green are fungal viruses from the unclassified group described by (Myers et al. 2020) and are labelled as in the original publication. Sequences shown in grey are from previous large-scale virus discovery research projects—either serratus (Edgar et al. 2022) (labelled as serratus) or RdRp-scan (Charon et al. 2022) (labelled as charon2022). Node labels represent branch support. (b) Density plot showing the length distribution of fungal DNA source uncharacterized proteins classified in this clade in comparison to proteins from ICTV-classified Durnavirales. (c) Tanglegram comparing the phylogenetic relationships of Mortierellaceae fungi (based on Vandepol et al. 2020), and those of the most common clade of durna-like viruses identified in each taxon. Colours correspond to Vandepol clusters and prefixes to the isolates labelled in the same publication. Node labels represent branch support.

In the *Durnavirales* order of dsRNA viruses, 107 EDRdRps and 19 EMRdRps were identified. Seventy-one of these 126 sequences are from members of the Mucoromycota division of fungi. Of these 71, 64 fall within a single clade, shown in Fig. 3a, and are found in the Mortierellaceae family of fungi. The mean length of sequences in this clade is 646 amino acids, compared to 1495 amino acids for ICTV classified *Durnavirales* (Fig. 3b). The closest ICTV classified virus to these putative EVEs is Curvularia thermal tolerance virus, a member of the *Curvulaviridae* family of viruses. Its host, *Curvularia,* is a member of the Pleosporaceae family of fungi.

Our Mucoromycota group forms a separate monophyletic group to this virus and its relatives, while several previously identified but unassigned viruses in Mucoromycota, particularly relatives of the bipartite mycoviruses identified by Myers et al. (2020), do fall within this newly identified group, as do a number of sequences from other virus discovery projects (Fig. 3a).

Of the uncharacterized protein sequences we identified in this clade, almost all (53/64) are from the same publication, which investigated the phylogeny of the Mortierellaceae (Vandepol et al. 2020). No viruses or EVEs were discussed in this publication, which focused on host phylogenetics. However, of the 316 fungal isolates sequenced in this paper, 53 included contigs with members of this putative EVE family.

Figure 3c compares the host phylogeny from Vandepol et al. (2020) with the relationships between the EVEs. The two trees are much more similar than those for the mito-like viruses (Fig. 2e), and the majority of the host tree can be recapitulated using the EVE tree. This suggests either a much longer-standing relationship between these viruses and their hosts, with EVEs inserted prior to the last common ancestor of the members of this family, or a very strong host specificity of the viral strains. The sequences within the clade are relatively diverse, with a mean similarity of 66%; this again suggests that these EVEs may be ancient.

#### Reovirales

*Reovirales* EVEs are not particularly well-known, but have been previously identified in mosquitoes (Palatini et al. 2022). We found a number of EVEs related to the *Reovirales*, specifically to proposed (but not ICTV-classified) members of the *Orbivirus* genus, in a well-supported clade containing primarily exogenous insect viruses (Fig. S5d). Most of the EVEs we identified in this clade amongst our uncharacterized proteins are also from insects, with two sequences from the Edith’s checkerspot butterfly (*Euphydryas editha*), one from the Mexican paper wasp (*Mischocyttarus mexicanus*), one from the cabbage stem flea beetle (*Psylliodes chrysocephala*), and five from the Asian citrus psyllid (*Diaphorina citri*). These diverse insect insertions suggest that endogenous *Reovirales* are more widespread amongst insects than has previously been thought, especially within this specific virus clade.

Interestingly, five EVE sequences were also found within this virus clade amongst uncharacterized proteins from two nematode species—the parasitic roundworms *Brugia pahangi* and *Brugia malayi* (Fig. S5d)*.* These worms are from the Onchocercidae family of filarial nematodes. Both are pathogenic; *B. malayi* causes lymphatic filariasis in humans, and *B. pahangi* causes filarial disease in cats and dogs, with occasional zoonotic transmission to humans (Bhumiratana et al. 2023). Both are vectored by various species of mosquito (Mulyaningsih et al. 2019). Historically, the RNA virome of nematodes has not been well known; however, a number of new viruses were identified in a recent virus discovery project (Quek et al. 2024). This publication identified two *Reovirales*-like sequences in nematodes: one a member of the Onchocercidae family, *Onchocerca ochengi*, a filarial (but not lymphatic) nematode of cattle closely related to *Brugia,* and one in *Haemoconchus contortus*.

**Figure 4:**
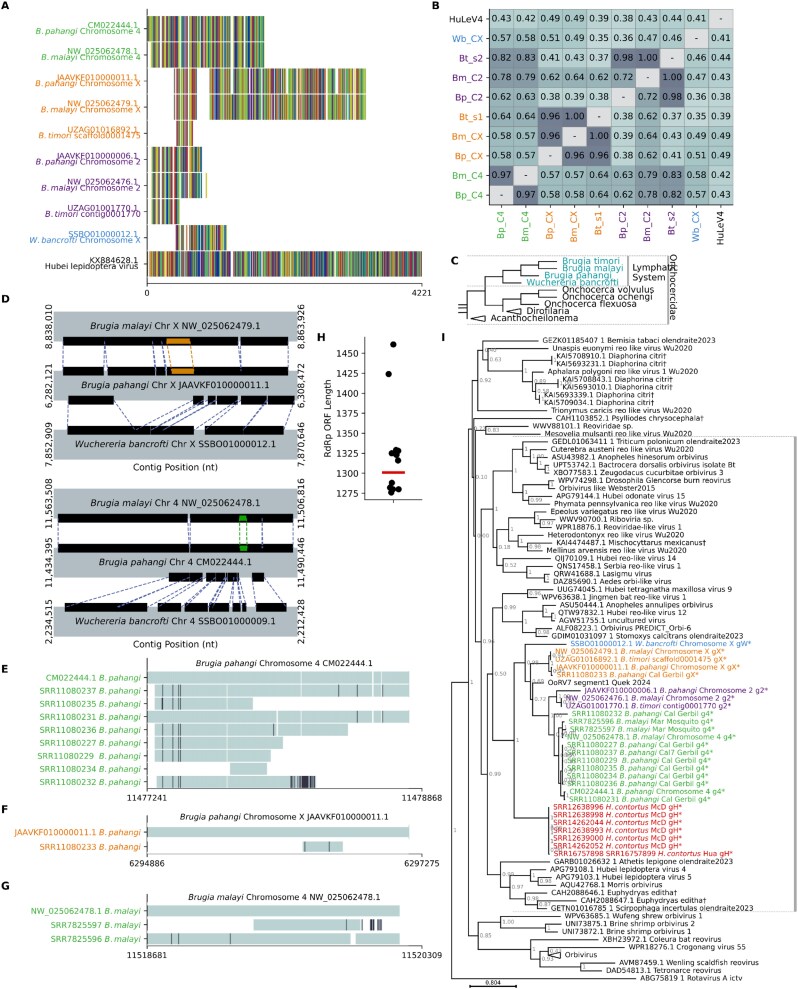
Orbi-like EVEs. (a) CIAlign (Tumescheit et al. 2022) mini alignment showing the regions of the RdRp gene represented in chromosomal regions of *B. pahangi*, *B. malayi*, *B. timori,* and *W. bancrofti* with similarity to orbi-like RdRp. Hubei lepidoptera virus 4 provides a full-length reference RdRp. Text colours correspond to clusters based on genome position. (b) Similarity matrix showing the proportion of identical nucleotide sites (excluding gaps) between the aligned EVE regions shown in (a). Abbreviations: *B. pahangi*, bp; *B. malayi*, bm; *B. timori*, Bt; *W. bancrofti*, Wb; chromosome, C; Hubei lepidoptera virus 4, HuLeV4. (c) Simplified phylogenetic tree for the Onchocercidae, generated manually based on Qing et al. (2021). Clades for genera not discussed in the text are collapsed. (d) Schematic showing the regions surrounding the orbi-like EVE insertions on *Brugia* and *Wuchereria* chromosomes X (top) and 4 (bottom). Regions shown in black and linked by dotted lines were successfully aligned using NUCMER (Kurtz et al. 2004). Coloured regions represent the position of the EVE. (e–g) CIAlign mini alignments showing the sections of SRA assembled contigs that aligned to *B. pahangi* chromosome 4 (e), *B. pahangi* chromosome X (f) and *B. malayi* chromosome 4 (g). Teal regions are identical; white represents gaps and black represents differences from the chromosome. Alignments for (e–g) are available in the supplementary data. (h). The distribution of ORF lengths of previously annotated RdRps in viruses in the marked clade (black dots) compared to those identified in the *H. contortus* contigs (pink line). (i) Approximate maximum likelihood (Price et al. 2010) amino acid phylogenetic tree for the newly identified orbi-like exogenous and endogenous viruses from nematode (labelled with *) and previously annotated sequences in this clade. Newly identified sequences are coloured by cluster and given a corresponding suffix—Green, g4, chromosome 4-like; orange, gX, chromosome X-like; purple, g2, chromosome 2-like, red, gH, *Haemonconchus*-like; and blue, gW, *Wuchereria*-like. Prefixes are GenBank or SRA accessions except for sequences with the suffix Wu2020, which are from Wu et al. (2020). The ‘true’ *Orbivirus* clade has been collapsed and contains a monophyletic group that incorporates all ICTV-classified members of the *Orbivirus* genus. The clade marked with a grey line is the orbi-like clade referred to in the text. FASTA files containing contig sequences and ORFs for newly identified sequences are available in the supplementary data, as is a Newick-formatted version of the phylogeny. Node labels represent branch support. A rotavirus A sequence was used as an outgroup.

On further interrogation based on our initial finding in *Brugia*, several regions resembling the orbi-like RdRp were identified. In both species, it was possible to identify a ~2400 nt region of chromosome X, a 1600 nt region of chromosome 4, and a 700 nt section of chromosome 2 resembling the *Orbivirus*-like (orbi-like) *Reovirales* (Fig. 4a). There are no ICTV-classified viruses within this clade, so the full RdRp sequence of Hubei lepidoptera virus 4 (KX884628.1) was used as a full-length reference. This sequence is 4041 nt in length, so the chromosome X region in *Brugia* is ~60% full length. All of the identified regions are highly degraded and do not contain the expected long RdRp ORF; instead, they contain many stop codons and frameshifts, typical of comparatively ancient endogenous insertions (Katzourakis et al. 2005).

*Brugia* EVE sequences were found to share far higher similarity with regions from the same chromosome in the other *Brugia* species than with insertions on other chromosomes within the same species, indicative of three separate insertion events. The chromosome X sequences from *B. pahangi* and *B. malayi* are 96% identical to each other on a nucleotide level, the chromosome 4 sequences are 97% identical, and the chromosome 2 sequences are 98% identical (Fig. 4a and b). Meanwhile, the three *B. pahangi* sequences share 38%–62% nt identity with each other and the three *B. malayi* sequences 57%–79%.

Using TBLASTX against the 229 available NCBI genomes from the phylum Nematoda, fragments resembling the orbi-like RdRp were identified in two additional species, *Brugia timori* and *Wuchereria bancrofti*. These species are also members of the Onchocercidae and are the closest known relatives of *B. pahangi* and *B. malayi* (Qing et al. 2021, Junsiri et al. 2024, Fig. 4a and c). They also both cause lymphatic filarial disease and are vectored by mosquitoes. Together, these four species represent all known lymphatic filarial worms. Two distinct *B. timori* sequences were identified, one shorter than but highly similar to the chromosome X insertions in the other *Brugia* species (96%–100% identity over 216 nt) and one to the chromosome 2 insertion (98%–100% identity over 380 nt) (Fig. 4a and b). The *W. bancrofti* insertion is also short (664 nt) and is not very similar to any of *Brugia* insertions, with nucleotide identities ranging from 35% to 58% (Fig. 4a and b).

Given that the sequences on the same chromosomes in *B. pahangi* and *B. malayi* share high similarity, the positions of the sequences on the chromosomes were examined to establish if they share an integration site and therefore are likely to have integrated into the genome prior to the divergence of the hosts from their last common ancestor. The chromosome 4 and chromosome X insertions were found to be at the same position in their respective genomes, within much longer homologous regions of the chromosomes (Fig. 4d). The *B. pahangi* and *B. malayi* chromosomes align closely, giving strong evidence that these insertions occurred before the divergence of the two species. This kind of alignment was not possible for the chromosome 2 insertions, which may therefore be at different sites or within more divergent regions. Integration prior to the divergence of these host species is consistent with the fact that the sequences cluster by chromosome rather than by host (Fig. 4b). The sections of RdRp that are present in the three species also fit with this hypothesis—the chromosome 2-like and chromosome 4-like insertions all contain the 5′ region of RdRp (relative to Hubei lepidoptera virus 4), while the chromosome X-like insertion is more central (Fig. 4a).

It was not possible to perform chromosome-to-chromosome alignment for *B. timori*, as both EVEs were identified at the end of short contigs and the *B. timori* genome is very fragmented. Previously, phylogenetic analysis has shown *B. timori* to share a more recent common ancestor with *B. malayi* than these two species share with *B. pahangi (*Qing et al. 2021, Junsiri et al. 2024, Fig. 4c, the date of this ancestor is not established). If this is the case, the insertions that are at the same position in *B. malayi* and *B. pahangi* must also be at the same position in *B. timori*.

The *W. bancrofti* sequence is also on chromosome X but is divergent from the chromosome X insertions in the other three species. It was possible to align chromosomes X and 4 of *W. bancrofti* with those of *B. pahangi*; however, the insertions did not occur within recognizable regions of similarity (Fig. 4d). No sequence resembling RdRp was identified on *W. bancrofti* chromosome 4, but there is a gap in the alignment at the position containing the insertion in *B. pahangi*, so it is not clear if the absence is due to poor sequencing of this region. For chromosome X, the alignment is also fragmented, but the chromosome X region resembling RdRp in *W. bancrofti* (positions 20 061 483–20 062 162) is distant from the region that aligned to the areas surrounding the insertion in *B. pahangi* (positions 6 282 121–6 308 472). Given the low similarity between the *W. bancrofti* sequence and the *Brugia* sequences (Fig. 4b) and the different region of the RdRp that is represented (Fig. 4a), it is likely to represent a different insertion event. If the insertions are not at the same positions in *W. bancrofti*, this means that their insertion date was a maximum of approximately four to six MYA, the estimated date of the last common ancestor of *Wuchereria* and *Brugia* (Small et al. 2014).

To identify if any of these EVEs are transcribed, publicly available RNA-seq datasets from nematodes containing putative *Reoviridae* RdRp-like sequences were identified using the Serratus project database (Edgar et al. 2022) and assembled. The resulting contigs were then screened using BLAST and HMMER to identify RdRp-like regions. Regions were identified in 18 datasets: 9 from *B. pahangi*, 2 from *B. malayi*, and 7 from *Haemonchus contortus* (there are currently no publicly available RNA-seq datasets in SRA for either *B. timori* or *W. bancrofti*).

The *B. pahangi* and *B. malayi* sequences identified in SRA datasets do not contain intact ORFs and instead resemble specific endogenous insertions. For *B. pahangi*, eight of the nine contig regions are highly similar to the chromosome 4 *B. pahangi* insertion and include only sections of the EVE that are present on chromosome 4 (Fig. 4e). The remaining *B. pahangi* contig region is identical to a short section of the chromosome X locus (Fig. 4f). The two *B. malayi* contigs appear to be derived from the chromosome 4 *B. malayi* locus, as again the ORFs are degraded and the region detectable in the contigs does not extend beyond the region encoded in the EVE (Fig. 4g). Therefore, it appears that the chromosome 4 and chromosome X insertions are at least sometimes transcribed in *Brugia*.

The other species in which orbi-like transcripts were detected, *H. contortus,* is a comparatively distant relative of the lymphatic filarial nematodes. It is of the same order, Rhabditida, but it is not part of the filarial nematode (Filarioidea) superfamily. Instead, it is a pathogenic nematode of ruminants, commonly known as the barber’s pole worm, causing the disease haemonchosis (Besier et al. 2016). *Haemonchus contortus* is not mosquito-transmitted. The divergence between *Brugia* and the clade containing *Haemonchus* is estimated to have occurred 240 MYA (Rota-Stabelli et al. 2013), and there are many species that are more closely related to *Brugia* that showed no evidence of orbi-like transcripts or EVEs.

Unlike the *Brugia* contigs, the contigs derived from RNA-seq in *H. contortus* show hallmarks of active, circulating viruses. The genome of *H. contortus* has been sequenced and assembled at the chromosome level (GCA_000469685.2) but does not contain any regions resembling reoviral RdRp. Four of the SRA assembled contigs have a complete, 1301 amino acid ORF, comparable to exogenous viruses from this clade (Fig. 4h, Supplementary Data). It would be unusual (although possible) for an EVE to maintain an ORF of this length, capable of producing intact viral proteins, for any period of time. For the remaining three sequences, the ORF is truncated at the 3′ end, likely because the contig assembly is incomplete. The ORF makes up 96%–98% of the full contig length, again consistent with known, related viruses. These results are consistent with transcription from an intact, circulating RNA virus, rather than an EVE.

Phylogenetic analysis was performed incorporating the *H. contortus* sequences, reconstructed ORFs from the endogenous insertions in *B. malayi, B. pahangi, B. timori,* and *W. bancrofti* and the related SRA sequences and *Orbivirus* and orbi-like reference sequences (Fig. 4i). Our nematode sequences fall within a single clade with branch support of 100%, together with the *O. ochengi* sequence from Quek et al. (2024) but surprisingly not the *H. contortus* sequence from this paper.

As expected from the results above, the *Brugia* sequences cluster phylogenetically by chromosome rather than by host, with distinct, well-supported chromosome 2, chromosome 4, and chromosome X clades. The chromosome 2 and chromosome 4 insertions and their associated SRA transcripts cluster closely together, while the chromosome X insertions are somewhat more distant (Fig. 4i). The *H. contortus* sequences form a separate, monophyletic group from the sequences from filarial nematodes. The phylogenetic relationships in this tree suggest that the nematode orbi-like viruses share a common ancestor more recently with each other than they do with the insect orbi-like viruses. However, the virus phylogenetic tree reflects several independent endogenization events.

If the virus detected in *H. contortus* is exogenous, it is expected that sequences resembling the other orbi-like segments besides RdRp will be present in these datasets. Members of the ICTV-classified *Orbivirus* genus have 10 segments and encode 12 proteins (Roy 1996). However, some segments have never been identified for members of the phylogenetic clade containing the nematode viruses (marked on Fig. 4i). For viruses in this clade, all identifiable proteins resemble VP1 (RdRp), VP3, VP4, or NS1.

VP1 (RdRp), VP3, and VP4 could be identified amongst *H. contortus* contigs from all seven SRA samples. Phylogenetic trees based on VP3 and VP4 are similar to the tree based on VP1 (Fig. 4i, Fig. S7). The VP3 and VP4 ORFs are consistent with the length of those identified for other members of the clade (Fig. S7). Unidentified proteins from other viruses in this clade were also compared to the *H. contortus* contigs; however, no convincing similarity was found.

Segments from members of the *Orbivirus* genus are known to have common terminal sequences, usually 6 nt in length, which are somewhat conserved within species (Matthijnssens et al. 2022). However, these could not be found either for the *H. contortus* contigs or for any previously identified members of this clade, possibly due to incompleteness of the contigs representing the segments.

### Virus-labelled sequences

**Figure 5:**
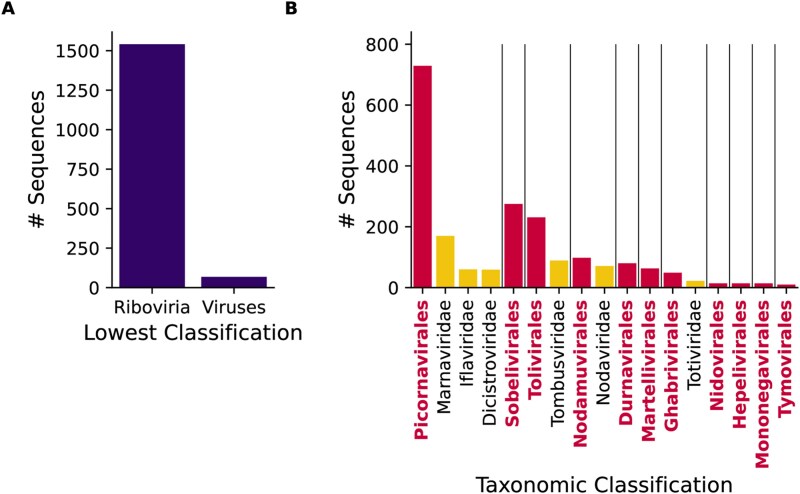
Virus-labelled sequences. (a) Bar chart showing the number of uncharacterized proteins with a previous lowest taxonomic classification of Riboviria or viruses. (b) Bar chart showing the ICTV RNA virus orders (pink, bold) and families (yellow) to which the virus-labelled uncharacterized proteins could be assigned. Each section shows families within the named order.

We identified 1609 uncharacterized proteins that are already classified as viral in the NCBI but not specifically as either *Orthornavirae* or RdRp. For 1541 sequences, the lowest taxonomic classification is the realm ‘*Riboviria*’ (NCBI Taxonomy ID 2559587), which includes not only the *Orthornavirae* kingdom but also the *Pararnavirae*, which encode RT rather than RdRp and several groups of satellite viruses (Fig. 5). The remaining 68 sequences are only classified at the superkingdom level, as ‘Viruses’ (NCBI Taxonomy ID 10239). Classifications are provided in Supplementary Table 1. These sequences are RdRp proteins from RNA viruses that either have not been officially taxonomically classified or that were yet to be classified at the time of their submission to the database. They are not labelled as RdRp but as uncharacterized proteins; however, all meet the criteria used here to classify a protein as a full or partial RNA viral RdRp. In many cases, these sequences were assigned to a taxon in their source publication, but this is not reflected in their database record.

Where possible, these sequences were provisionally assigned here to an ICTV-classified family. Only sequences that fell within the existing diversity of a well-supported (branch support ≥0.8), monophyletic cluster of ICTV-classified viruses from the same family were assigned to that family. This is a conservative approach that may cause more divergent members of the same family to be excluded. Using this approach, 510 viruses were classified at the family level. The largest groups were the Marnaviridae, with 170 members, Tombusviridae with 89, and Nodaviridae with 71 (Fig. 5b). We putatively assigned 1605 sequences to viral orders, most commonly the Picornavirales (729 sequences), Sobelivirales (275 sequences), or Tolivirales (231 sequences) (Fig. 5b).

### Sequences misclassified as bacterial

Amongst sequences classified as bacterial, 251 RdRp-like sequences were identified (Fig. 1b). Of these, 96 are almost identical sequences from DNA contigs assembled from the *Mesorhizobium* species of nitrogen-fixing bacteria (Supplementary Data: Tree Cryppavirales_11). These sequences are all from the same study into root nodules from chickpeas (*Cicer arietinum*) and have a highly significant TBLASTN hit (99% identity across 100% of the protein) against chickpea chromosome 7 (AHII03000007.1), so it is very likely that these represent an EVE from the host of the bacteria that has been misclassified.

**Figure 6:**
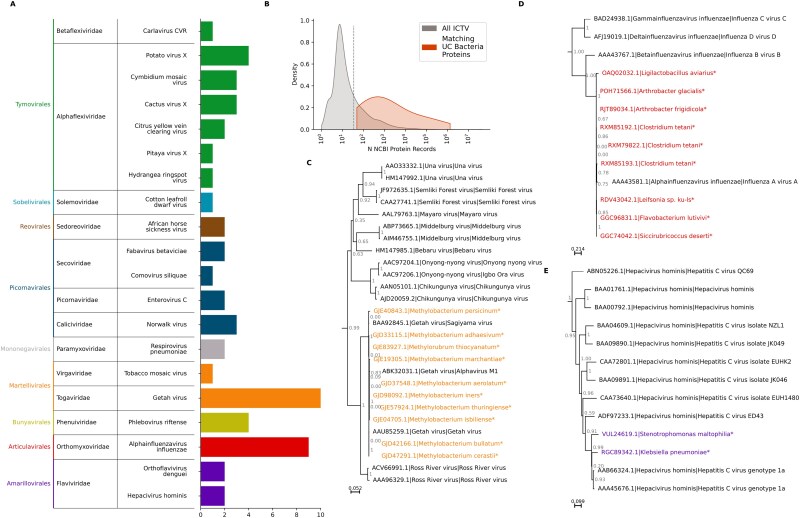
Sequences mislabelled as bacterial. (a) Bar chart showing, for sequences labelled in their original database as bacterial and sharing > 80% BLAST identity and a bit score > 400 with an ICTV-classified virus, the number of sequences resembling each indicated virus. Colours and labels in the left column represent viral orders. (b) Density plot comparing the number of GenBank records per species for all ICTV-classified viruses (grey) and for those which matched a bacterial-labelled RdRp-like uncharacterized sequence (red). (c–e) Approximate maximum likelihood (Price et al. 2010) amino acid phylogenies for specific examples of bacterial-derived sequences and previously identified sequences in the same clade, for sequences related to Getah virus (c), influenza virus a (d), and hepatitis C virus (e). Node labels represent branch support. Previously annotated sequences are labelled with their GenBank accession, then ICTV binomial species name, then common name. Tree files for (c–e) are available in the supplementary data.

Of the remaining RdRp-like unclassified sequences labelled as bacterial DNA, unexpectedly, 56 sequences had very high BLASTP similarity scores (>85% identity and bit score > 400) against diverse ICTV-classified RNA virus RdRps (Fig. 6a, Supplementary Table 1). All of the 56 sequences were found to match very commonly studied pathogenic viruses, including widespread pathogens of humans and agriculturally important animals and plants, such as the influenza A, dengue, hepatitis C, and African horse sickness viruses (Fig. 6a). As a proxy for how widely studied a virus is, the number of NCBI protein records was calculated for all ICTV-classified RNA viruses and compared to the number for ICTV-classified RNA viruses with matching ‘bacterial’ proteins (Fig. 6b). ICTV-classified RNA viruses have a median of 10 protein sequences available, while those with matching bacterial proteins have a median of 778. This supports a hypothesis that the sequences labelled as bacterial are the result of either mislabelling or contamination with common laboratory virus strains.

Phylogenetic analysis further supported this hypothesis, with the bacteria-labelled sequences indistinguishable from known pathogens. For example, ‘bacterial’ sequences were identified that fell well within the known diversity of *Alphavirus getah* (Getah virus) (Fig. 6c), *Alphainfluenza virus A* (Influenza A virus) (Fig. 6d), and *Hepacivirus homininis* (Hepatitis C virus) (Fig. 6e).

It is possible that these sequences are viruses incorporated into bacterial plasmids as part of vector constructs and labelled according to the bacterial rather than the viral taxon. However, only 2 of the 56 viral pathogen sequences are labelled as *E. coli*, the most common vector species. The other genera with which they were labelled, which were most commonly *Methanobacterium*, *Klebsiella*, *Xanthomonas,* and *Streptococcus*, are not routinely used in virology research.

The source nucleotide contigs from which the viral proteins were predicted were examined to further verify if they are likely to be part of vector sequences. In 54 of the 56 cases, the whole contig contains only RNA viral proteins (Fig. S8). The remaining two cases, two identical contigs uploaded by the same research group (JRJL01000069.1 and QNPM01000069.1) contain both bacterial and viral sequences from the vector pGR106 (Lu et al. 2003), which contains the Potato virus X genome and material derived from *Agrobacterium*. However, the contigs from which these sequences are derived are labelled as the bacterium *Xanthomonas oryzae* pv. *oryzae* (Xoo), which is unrelated to this plasmid. On further investigation, a 93 nt identical region was identified in the Xoo genome (CP050114.1, positions 459 812– 459 904) that shares 85% identity with the reverse complement of part of pGR106 (AY297842.1, positions 328–424). Therefore, the viral sequences adjacent to these bacterial sequences are likely to be the result of misassembly at this region of high sequence similarity.

These results together suggest that these are likely sequences from exogenous viruses that are the result of contamination of bacterial samples or sequencing flow cells with viral RNA, misassembly, or mislabelling of RNA viral samples as bacterial.

### Near-misses

**Figure 7:**
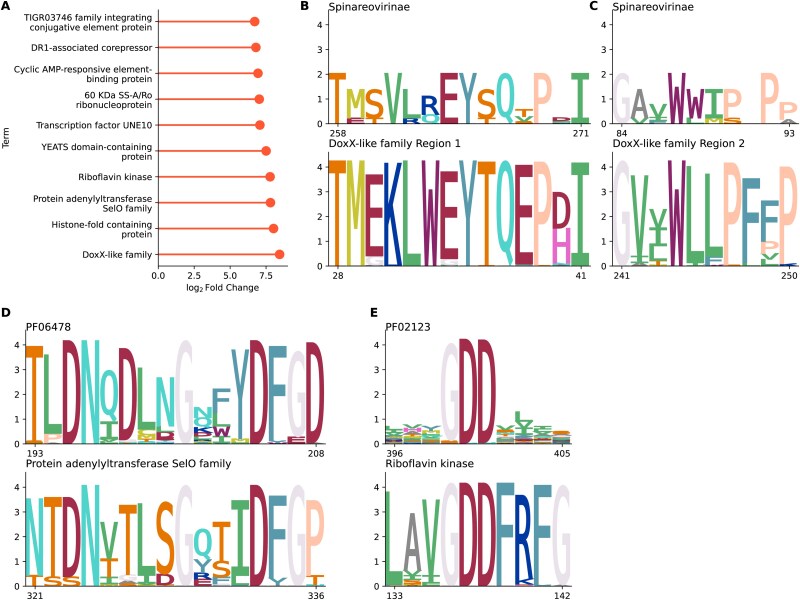
Near-miss analysis. (a) Bar chart showing the 10 terms that were most overrepresented amongst BLAST hits against uncharacterized proteins scoring between 10 and 25 in HMMER (Eddy 2011) comparisons with viral RdRp pHMMs compared to uncharacterized proteins with no score. Bars represent the log2 fold change between the frequency of the term in the two sets. (b–e) sequence logos generated with CIAlign (Tumescheit et al. 2022) showing the alignment between specific regions of the near-miss proteins and the pHMM profiles against which they had a weak match, for DoxX-like family proteins (b, c), protein adenylyltransferase SelO family proteins (d), and riboflavin kinase proteins (e). Positions in the near-miss proteins are relative to the alignments provided in the supplementary data; positions in the profiles are relative to the original profile.

Another aim of this study was to identify proteins that are not RdRp but that score highly in HMM-based comparisons with RNA viral RdRp. To this end, over 1 million uncharacterized proteins were examined that had an HMMER domain score against a viral RdRp profile that was ≥10 and ≤25, described hereafter as ‘near-miss’ proteins. These were compared to a randomly selected control group of uncharacterized proteins for which HMMER did not return a score against RdRp. The likely functions of both groups of proteins were determined using BLASTP against proteins of known function, and terms associated with these functions were categorized into groups. Of the near-miss proteins, 577,704 had an identifiable probable function, and these were compared with 499 240 identifiable proteins from the control group. Figure 7a shows the 10 terms that were most significantly overrepresented amongst the near-miss proteins compared to the control (also listed in the Supplementary Data).

Protein-related members of the DoxX-like family of integral membrane proteins were particularly overrepresented; 785 of the near-miss uncharacterized proteins matched proteins annotated with this term, compared to two of the control proteins. The BLAST target sequences most similar to these proteins were 191 closely related proteins, ~300 amino acids in length, from the *Bacillus* genus of bacteria. An MSA for these sequences is available in the Supplementary Data. Two distinct regions were identified, resembling the HMM profile for the *Spinoreovirinae* RdRp, labelled here as region 1 and region 2 for clarity (Fig. 7b). The region 1 domain (positions 23–76 in the MSA) has similarity to part of the StAR-Related Lipid-Transfer (START) domain, while the region 2 domain (positions 239–271 in the MSA) does not match any specific domain of the DoxX-like proteins. Lipid transfer proteins do play a role in the life cycle of some RNA viruses (Avula et al. 2021), and it is interesting that both regions match specifically the *Spinoreovirinae*, but there is not enough evidence here to infer anything other than coincidental similarity. Nevertheless, DoxX-like proteins could be incorporated into negative control protein sets to test the specificity of RdRp-detection methodologies.

A moderate level of similarity to the Pfam profile for Coronavirus RNA-dependent RNA polymerase, N-terminal (PF06478) was identified amongst unidentified proteins with similarity to 56 bacterial proteins, mostly from the genus *Corynebacterium*, annotated as protein adenylyltransferase SelO family proteins (MSA provided in Supplementary Data, high similarity was detected at positions 297–348). These are pseudokinase proteins involved in cellular response to oxidative stress (Sreelatha et al. 2018). Again, there is no particular reason to suspect these proteins are related, but there is visible similarity between the PF06478 RdRp and the SelO proteins within a specific region (Fig. 7c).

A set of riboflavin kinase genes from various bacterial genomes also shows some similarity to the near-miss proteins (MSA provided in Supplementary Data, high similarity was detected at positions 132–168). In this case, the conserved GDD motif that is common in RNA viral RdRp (Koonin 1991) is apparent in the bacterial alignment.

### Negative control sets

Two curated sets of likely non-RNA viral uncharacterized proteins were generated as part of this study and are available as negative controls for future studies. Both sets contain uncharacterized proteins from the NCBI Protein database that meet the following criteria: at least five significant BLASTP hits (bit score > 50, percentage identity > 40%, alignment length > 100) against characterized, non-RNA-viral proteins; no BLASTP hits meeting these criteria against characterized RNA viral proteins; at least one CDD annotation but none that are found exclusively in RNA viruses; and no PalmScan hits against RdRp. The negative control (NC) set contains 20 000 uncharacterized proteins that meet all of these criteria and additionally had no HMMER results with a score ≥ 10 against any of the RNA virus RdRp pHMMs described above. The near-miss (NM) set contains 20 000 uncharacterized proteins that meet all of the initial criteria but have an HMMER domain score ≥10 and <25 against an RNA virus RdRp pHMM. These datasets are provided as Supplementary File 1 (NC) and Supplementary File 2 (NM).

## Discussion

By screening only the uncharacterized proteins in the NCBI Protein and UniProt databases for a single viral gene, a surprising number of RNA viral RdRp-derived proteins, particularly RNA viral EVEs integrated into eukaryotic genomes, were identified. These results both reveal the extensive diversity of unknown EVEs and how they can impact our understanding of contemporary viruses and demonstrate the complexities of generating negative control datasets for RNA virus discovery. Several of these newly identified RdRps provided starting points to investigate the host range and diversity of different viral taxa.

It is clear from our results that non-RNA viral proteins from the NCBI protein or UniProt databases, used without additional filtering, are not a good negative control in RNA virus discovery research, as they contain ‘false false positives’—sequences that will show as false positives (RdRps in the negative control) but that are actually mislabelled true positives. The 3560 proteins described here were all identified as RNA viral RdRp *via* at least two sources of evidence and fall comfortably within RNA viral phylogenies. It is therefore recommended that, if these databases are used to eliminate potential false positives when searching for RNA viral RdRp, uncharacterized proteins are first removed. Additional putative false positives found amongst named proteins should be carefully checked to ensure that the named proteins are not from an RNA viral or EVE source.

As the requirements of a negative control set vary by study, we also present a set of uncharacterized proteins that have been filtered for similarity to RNA viral RdRp based on BLAST, HMMER, CDD, and PalmScan annotations. This set is not intended to be comprehensive—proteins with no similarity to anything in this set cannot be said to be definitively viral—but to provide a likely RdRp-free set of diverse proteins as a negative control for newly developed RdRp detection software and to provide an additional training set for machine learning models. Adding these proteins will help avoid some potential pitfalls of excluding uncharacterized proteins, such as biases towards common proteins that are more likely to be fully characterized and towards well-annotated organisms. Our set of ‘near-miss’ target proteins, named proteins that are not RdRp but do share some sequence similarity with RdRp, can also be incorporated as negative controls when developing RdRp detection software, as they represent a likely source of genuine false positives.

Besides their utility in future methods development, the results presented here also show that EVEs remain under-studied and undercharacterized. This study had a relatively limited scale compared to much virus discovery research but uncovered hundreds of previously unknown EVEs. Plants and insects, in particular, were a rich source of EVEs from diverse virus families. EVEs were also found in unexpected taxa, for example, the Perkinsozoa phylum of protists and the parasitic worm *P. laevis*, for which almost nothing is currently known about the RNA virome.

Our findings for the *Cryppavirales* are consistent with the currently accepted hypothesis that plant *Mitoviridae* viruses share a more recent common ancestor with each other than with *Mitoviridae*-infecting fungi (Bruenn et al. 2015, Nibert et al. 2018, Jacquat et al. 2023), in that our plant mito-like viruses fall almost exclusively in a single clade within the Duamitovirus genus. *Mitoviridae* in plants are considered to have initially integrated in the mitochondria and later been passed to the nuclear genome by DNA transfer (Bruenn et al. 2015). Around one in four of our sequences were identified in mitochondria, but many additional sequences are from nuclear or unassigned contigs. Identical or near-identical mitochondrial and nuclear sequences are common amongst our results. This suggests that, at least in some cases, if the nuclear EVEs are integrated mitochondrial DNA, then the integration has occurred recently, which is feasible since the process of mitochondrial transfer of DNA into the nucleus is considered to be ongoing in plants (Zhang et al. 2020). Older insertions into the nuclear genome would be expected to have degraded to some extent. Based on our results, integration of mitoviral EVEs seems to be diverse and widespread across the Magnoliopsida class of flowering plants.

Other EVEs identified here appear to be much more ancient. For example, the large cluster of fungal viruses related to the Curvulaviridae (Fig. 3a) contains likely exclusively endogenous sequences with which the host phylogeny can be almost fully recapitulated. It is likely that the viruses found here are older than the divergence of this group from a common ancestor and that if further members of this family were subjected to genomic sequencing, more examples would be identified.

The results for the orbi-like viruses present an interesting case, with exogenous and endogenous viruses in specific, distantly and closely related nematode species. For the *Brugia* orbi-like viruses, there is no evidence of ongoing exogenous viral activity; however, there have been at least three separate integration events, seemingly prior to the divergence of the species of *Brugia* from their common ancestor. The EVEs in the genome appear to be ancient and are moderately degraded. However, the virus identified in *H. contortus* shows hallmarks of being a currently circulating, exogenous virus, suggesting ongoing activity by viruses in this clade throughout its evolutionary history. The fact that these closely related viruses were identified in both *Haemonconchus* and the filarial nematodes, despite their large genetic distance, implies both that there are likely additional orbi-like viruses still to be discovered amongst nematodes and that nematodes may be common hosts for members of this clade.

The results presented here have been identified fully computationally and based only on publicly available sequences, and therefore, some limitations are inherent. The metadata attached to the original sequences can be erroneous or incomplete, leading to misclassification of the host species. It is also not possible to say definitively if the host species of these viruses are correct, except for EVEs with clear flanking sequences. Using our approach, it is possible that some spurious matches to unrelated proteins remain amongst our identified putative RdRps. Further analysis of protein structures and transcripts, along with experimental validation, is required for definitive classification; meanwhile, the present results should be interpreted with these caveats in mind. It is also likely that other viral RdRps exist amongst the screened datasets that are beyond the limits of our detection protocol. New sequences are also added daily to both databases searched here, and new RNA viruses are constantly being discovered, so repeating the analysis would almost certainly uncover new results.

Classification of EVEs is complex, as, especially for highly degraded sequences, these elements may retain little or none of the function or phenotype of exogenous viruses. The classification of EVEs using taxonomy designed for exogenous viruses has been under debate for a number of years (Blomberg et al. 2009, Gifford et al. 2018). However, EVEs are derived from circulating viruses and can only be understood in the context of their exogenous relatives. Whether or not EVEs are classified as viral in the strict sense, their origins and relationships are best understood with reference to related, currently circulating viruses.

One issue highlighted by our analyses is that the metadata attached to database records commonly contains errors or is insufficient to determine the true provenance of a sequence. This is a common issue across genomic databases (Gonçalves and Musen 2019, Ritsch et al. 2023, Charon et al. 2024, Chorlton 2024). To some extent, errors are inevitable; however, database providers can do much to mitigate this by enforcing metadata standards such as Minimum Information about a high-throughput nucleotide SEQuencing Experiment (MINSEQE) (Brazma et al. 2012) are scrupulously followed. Allowing users to flag sequences with known issues would be valuable. There is also ambiguity about how complex sequences such as EVEs should be classified, an issue that would benefit from clearer guidance and standardization by database providers.

In conclusion, RdRp-like sequences, derived from both exogenous viruses and EVEs, are common amongst the uncharacterized proteins in public protein databases. Many EVE proteins in particular remain uncharacterized and provide a rich, largely untapped, source of data about virus host range and evolutionary history.

## Materials and methods

To identify uncharacterized and poorly characterized proteins, six search terms were used: uncharacterized, unclassified, unknown, unnamed, hypothetical, and DUF. These are referred to henceforth as the ‘unknown protein search terms’.

Proteins were included in the target database as follows: National Center for Biotechnology Information (NCBI) Protein database (Sayers et al. 2021) (accessed 2022-08-08) proteins containing any of the unknown protein search terms in their ‘title’ field and not classified taxonomically as ‘*Orthornavirae*’ and proteins from UniRef100 (Suzek et al. 2015) (accessed 2023-05-31, 2023-05-03 EBI release) that contain any of the unknown protein search terms or ‘putative’ in the protein name.

Three sets of publicly available pHMMs were selected to allow for comprehensive screening. Pfam profiles are based on alignments extracted from the HHsuite-3 pfamA 35.0 database (Steinegger et al. 2019) (accessed 2023-02-17, 2021-11-23 release), for 22 Pfam families identified as RNA viral RdRp, these are listed in the Supplementary Data. Transcriptome shotgun assembly (TSA) profiles are the 74 pHMMs described by Olendraite et al. (2023). RdRp-scan profiles are the 43 pHMMs described by Charon et al. (2022). All pHMMs were created from the provided multiple sequence alignments using HMMbuild from HMMER v3.3 (Eddy 2011).

pHMMs were compared with the target database using HMMscan from HMMER, with the default settings. HMMER results were filtered to retain only sequences with a domain score ≥ 25. For the near-miss analysis, results were filtered to retain sequences with a domain score ≥ 10 and < 25. RefSeq records were removed if they were copies of another sequence already in the database. Sequences present in the RdRp-scan database (Charon et al. 2022) and therefore already known to be RdRp were also excluded.

Details of filtering reverse transcriptase hits from Pfam profiles PF05919 and PF00680 are provided in the Supplementary Materials and Methods.

### Virus BLAST searches

All sequences passing the HMMER filtering stage were subjected to DIAMOND BLASTP (v0.9.14) (Buchfink et al. 2021) searches against the nonredundant BLAST protein database (nr, downloaded 2023-06-14), to further characterize the RdRp-like proteins. If there were no matches against characterized proteins with DIAMOND BLASTP, the search was repeated using BLASTP *via* the online NCBI BLAST server (https://blast.ncbi.nlm.nih.gov/Blast.cgi, accessed 2023-08-08) and the results processed as for the DIAMOND BLAST results. Full details of BLAST processing are provided in the Supplementary Materials and Methods. Supplementary Table 1 lists the best BLASTP match (excluding uncharacterized sequences), the BLAST methodology used (DIAMOND BLAST or online BLASTP), and the BLAST bit score against the best match for all analysed proteins.

### Clustering and initial taxonomic assignment

Proteins passing this initial filtering were classified into approximately order-level taxonomic groups based on the pHMM to which they had the highest scoring match and then smaller operational taxonomic units (OTUs) by clustering followed by rounds of multiple sequence alignment and phylogenetic analysis. Full details of this process are provided in the Supplementary Materials and Methods. Sequences were assigned to the OTU within which they had the shortest distance (calculated from the trees with the ete3 v3.1.2 (Huerta-Cepas et al. 2016) get_distance function) to any known virus. The OTU assigned to each sequence and its distance from the nearest neighbour within the tree are provided in Supplementary Table 1.

### Filtering HMMER results

To increase the probability that identified sequences are truly RNA viral, additional screening was performed with Foldseek, PalmScan, and BLAST and using the phylogenies generated above.

Sequences were classified as likely RNA viral if they met two of more of the following criteria (software details are provided in the Supplementary Materials and Methods): (i) A high FoldSeek bit score of >100 against an RdRp profile, (ii) an identified RdRp core using PalmScan, (iii) a high BLAST bit score of >50 against a known RdRp sequence in the BLASTP analysis described above, (iv) a branch length of <1.5 amino acid substitutions per site separating the sequence from the nearest known RNA virus in phylogenetic analysis, or (v) a HMMER domain score > 50 against an RdRp profile. PDB profiles used with FoldSeek were classified manually as RdRp based on the profile metadata. For BLAST searches sequences were compared to the whole of the NR database and then results filtered manually to include only those matching previously described RNA viruses or endogenous RNA viruses (based on the literature, NCBI taxonomy, NCBI protein metadata, and the GenBank record including a labelled RdRp), plus additional filtering as described in the Supplementary Materials and Methods. Known RNA viruses in phylogenetic analysis are those incorporated into the viral OTUs described in the Supplementary Materials and Methods.

Specific settings for each of these tools are provided in the Supplementary Materials and Methods.

### Phylogenetics

Phylogenetic trees for each viral OTU were regenerated, containing only the sequences passing these filtering criteria. To generate these trees, sequences were aligned with MAFFT (v7.520) (Katoh et al. 2002) under the default settings; then, alignments were cleaned with CIAlign (v1.1.4) (Tumescheit et al. 2022) to remove insertions up to 1000 amino acids in length, remove terminal regions with coverage < 10% or similarity < 10% and remove sequences < 50 amino acids in length. Trees were then generated with FastTree (v2.1.11) (Price et al. 2010), under the default settings. These trees are available in the Supplementary Data. These phylogenetic assignments are not intended to be definitive and should be combined with the BLAST and pHMM results to confidently assign RdRps to specific taxa.

Methodology used specifically to characterize the *Cryppavirales* and *Reovirales* sequences is provided in the Supplementary Materials and Methods.

### Virus-derived sequences

To classify identified proteins already labelled as viral, a BLASTP search (v2.14.0) (Altschul et al. 1990, Camacho et al. 2009) was performed for uncharacterized proteins from records labelled as ‘*Riboviria*’ or ‘Viruses’. These were compared to RdRp proteins linked from the nucleotide accessions listed in the ICTV virus metadata resource (version VMR_19-250422_MSL37) from 5000 viruses across 3905 defined species. The uncharacterized proteins were incorporated into phylogenetic trees containing all ICTV RdRps from the same viral order as their best-matching ICTV RdRp, by aligning sequences using MAFFT and then building trees using FastTree, both with the default settings. Monophyletic groups containing only uncharacterized proteins and viruses from a single ICTV classified order or family, with branch support ≥0.8 were used to make putative family- and order-level assignments.

### Near-misses

Details of the methods used to characterize the near-miss sequences are provided in the Supplementary Materials and Methods.

## Supplementary Material

supp_m_doc_clean_refs_veaf074

supplementary-material_veaf074

## Data Availability

Supplementary data have been deposited *via* Zenodo at https://doi.org/10.5281/zenodo.16993714. All raw data are from public repositories.
