# Supplementary material for: Uncovering hundreds of exogenous and endogenous RNA viral RdRp sequences amongst uncharacterized sequences in public protein databases
Source: Virus Evol. 2025 Sep 18;11(1):veaf074. doi: 10.1093/ve/veaf074 (PMC12548735; doi:10.1093/ve/veaf074)
Supplement: supp_m_doc_clean_refs_veaf074 [file supp_m_doc_clean_refs_veaf074.docx]

**Supplementary Materials and Methods**

**Filtering Reverse Transcriptase**

To remove hits against reverse transcriptase from the Pfam profiles PF05919 and PF00680, which contain both RdRp and RT, a phylogenetic tree was generated consisting of the sequences included in these profiles. The alignments used were extracted from the HHsuite-3 pfamA 35.0 database (Steinegger *et al.* 2019) (accessed 2023-02-17, 2021-11-23 release), and trees were generated with FastTree (v2.1.11) (Price *et al.* 2010), with the default settings. New profiles were created based on the phylogeny using HMMbuild from HMMER (v3.3) (Eddy, 2011), using subsections of the original alignment. PF05919 was divided into two subsections – RT and RdRp, while PF00680 was divided into RdRp, RT1 and RT2, as the RT section of the phylogeny had two clear subgroups of RT (Figure S1). Ambiguously placed sequences and long branches (length >3) were excluded from the profiles. Aligned FASTA files and HMMs for the newly created profiles are available in the Supplementary Data. Sequences which originally matched either PF05919 or PF00680 were compared to each of these profiles with HMMER as for the original profiles, and assigned as either RT or RdRp based on the higher HMMER score.

A few scattered sequences tagged as other proteins fell into the RdRp clades. For PF00680, A0A1B0C5W8, labelled as the phospholipid scramblase protein (PLSCR2) of the tsetse fly *Glossina palpalis,* was in the RdRp-like cluster (Figure S1A). For PF05919, A0A371IGY1, labelled as the velvet bean *Mucuna pruriens* NADH-ubiquinone oxidoreductase chain 6 (ND6) and A0A5A7QJR6, labelled as red witchweed *Striga asiatica* Photosystem I P700 chlorophyll a apoprotein A1 (PSAA), were in this group (Figure S1B). In all three cases, BLAST (Altschul et al. 1990, Camacho *et al.* 2009) searches showed different sections of the sequences were highly similar to either RdRp or to eukaryotic proteins, suggesting either endogenisation of viral sequences or misassembly. A number of unclassified sequences fell into the RdRp-like group in both cases (Figure S1).

**Clustering**

Proteins passing the initial filtering step were classified into approximately order-level taxonomic groups by clustering followed by multiple sequence alignment and phylogenetic analysis. Sequences were clustered using the MMseqs2 (v14.7e284) (Steinegger *et al.* 2017) cluster algorithm at 85% similarity and 25% coverage in cluster mode 0 and with ‘--cluster-reassign’ enabled. The representative sequences were aligned to the sequences making up the HMM profile to which they had the most significant match and provisional phylogenetic trees were generated, using MAFFT (v7.520) (Katoh *et al.* 2002), and then FastTree (v2.1.11) (Price *et al.* 2010), both with the default settings. Based on their clustering in this analysis, sequences were assigned to one of 32 virus groups based on the profile to which they matched. These groups are predominantly at order level, with the exceptions of the phylum Lenarviricota (for which many published sequences were not classified at a lower level), the families *Birnaviridae* and *Permutotetraviridae*, which do not have a higher level International Committee on Virus Taxonomy (ICTV) classification and the provisional genera Zhaovirus, Yanvirus and Weivirus (Shi *et al.* 2016) and Quenyavirus (Obbard *et al.* 2020), which have not yet been classified by the ICTV.

Within these order-level groups, existing viral sequences have been subdivided, based on our previous phylogenetic and clustering analyses, into the 331 operational taxonomic units (OTUs) as described below. Clusters of uncharacterised proteins were expanded into the original sequences and each sequence was sequentially incorporated into the phylogenetic tree for each OTU within its assigned order-level group, using the MAFFT --add function and FastTree with settings as above. Sequences were assigned to the OTU for which they had the shortest mean branch length to all other sequences in the OTU. Phylogenetic trees for the OTUs to which the sequences were assigned are provided in the Supplementary Data.

**Generating Viral OTUs**

Known virus sequences used to generate viral OTUs were identified from ICTV, RefSeq, NCBI Protein and 26 publications as listed in Supplementary Table 2. Additional known virus sequences were included from the reference databases used in these publications.

To access known RdRp sequences, where sequences had GenBank protein accession numbers, records were downloaded using the Entrez efetch tool (Kans 2013). Where GenBank nucleotide accessions were provided the associated protein accessions were pulled from the GenBank records. Where possible, RdRp was identified based on sequence annotations, otherwise, HMMScan (Eddy, 2011) was used on all protein sequences, from each nucleotide record, with the default settings, against the RdRp HMM databases from Olendraite *et al.* 2023 and Charon et al. 2022. The sequence with a HMMER score >40 which was most similar to any RdRp was assigned as the likely RdRp sequence. Where no RdRp proteins could be identified via either of these routes, all ORFs longer than 50 amino acids were identified in the nucleotide sequences using orfipy (Singh and Wurtele, 2021), allowing partial 3’ and 5’ sequences and stop-stop ORFs. These ORFs were then used as the input for HMMER, with the same settings, and again the sequence with a HMMER score higher than 40 which was most similar to any RdRp was assigned as the likely RdRp sequence. Sequences where no RdRp could be identified through any of these methods were excluded. Where no GenBank accessions were provided but nucleotide sequences were provided supplementary to the publication, these were used as input for the same process.

ICTV sequences are based on the ICTV Virus Metadata Resource (version VMR_19-250422_MSL37) and downloaded from GenBank. RefSeq sequences are based on searching NCBI RefSeq Protein for all sequences with taxonomy IDs 2732396 (*Orthonavirae*), 35325 (dsRNA viruses) and 2585030 (unclassified *Riboviria*) on 04/06/2023, then screening these sequences for RdRps using HMMER as above, again identifying ORFs with orfipy if no proteins were annotated.

An additional 1,137 sequences were added manually based from NCBI protein based on searches covering under-represented groups. In total, from all sources, 336,157 RdRp sequences are included in this database.

Supplementary Table 2 lists all sequence sources, along with download dates.

Sequences were then grouped at approximately taxonomic order level. Where records were available through NCBI and had a taxonomic assignment at order level, this assignment was used. Where this was not possible sequences were assigned manually by examining the phylogenetic analyses in their source publications or based on the annotations in supplementary data files provided with their source publications. If none of these methods were possible, the most similar pHMM in HMMER analysis was used to give a broad taxonomic classification.

Each order level group was clustered at 85% identity over 25% of the length of the sequence, using cluster mode 0 in MMSeqs (Steinegger *et al.* 2017). To improve alignment quality around the RdRp core, RdRp palmprint sequences were identified using PalmScan (Babaian and Edgar, 2022) and initially aligned for each group, using MAFFT with the linsi algorithm. Full length sequences were then added to these alignments using the MAFFT –add feature, and the shorter palmprint only sequences removed. Phylogenetic trees were built for each group using FastTree, with the default settings. Phylogenetic outliers, with branch lengths >5 from their nearest relative, were removed, this threshold was determined by examining the trees. Each tree was examined manually to ensure that expected phylogenetic relationships were apparent and that no visible outliers were present. After this process, 191,996 of the representative sequences from the first clustering pass remained.

Each of the resulting subgroups is considered here to be a viral OTU. Newly identified sequences were aligned to each OTU of the same order, again using the MAFFT –add feature, then assigned to the OTU within which they had the shortest mean distance (calculated from the trees with the ete3 v3.1.2 (Huerta-Cepas *et al*. 2016) get_distance function) to all other terminal nodes.

**Virus BLAST Processing**

DIAMOND BLASTP (v0.9.14) (Buchfink *et al.* 2021) searches against the non-redundant BLAST protein database (nr, downloaded 2023-06-14) were used to further characterise the RdRp-like proteins. If there were no matches against characterised proteins with DIAMOND BLASTP, the search was repeated using BLASTP via the online NCBI BLAST server (<https://blast.ncbi.nlm.nih.gov/Blast.cgi>, accessed 2023-08-08) and the results processed as for the DIAMOND BLAST results. BLASTP results were filtered to exclude self hits. The most similar ten target proteins not labelled as uncharacterised were identified. Sequences matching only RdRp were classified as “RdRp”; those matching RdRp and another adjacent named protein as “RdRp plus adjacent”; those matching reverse transcriptase, maturase or integrase as “RT-like”; and those matching only a named protein which is neither RdRp or RT as false positives. Target proteins were examined manually to ensure they were assigned to the correct category. Proteins classified as RNA virus or RNA virus plus adjacent were considered to be true positives, all others to be false positives. Proteins matching only uncharacterised proteins were retained but required a further line of evidence (as described below) to be classified as RdRp. Three named proteins in nr were found to be likely mislabelled RNA viral proteins, these were ACF19853.1 (ANT-5, *Toxicara canis*), KMQ93496.1 (gag-pol fusion protein, *Lasius niger* and KMQ91513.1 (glucuronate isomerase, *Lasius niger*), therefore matches against these three sequences were considered to be true positives.

**Filtering HMMER Results**

Foldseek analysis was performed to identify proteins with protein structural similarity to RdRp. Putative RNA viral proteins were split into substrings with a length of 400 amino acids and an overlap of 200 amino acids (as this is the maximum size which can be processed). These sequences were folded using the ESM Fold Sequence (Lin *et al.* 2023) online server (accessed via API on 2023-07-04 at <https://api.esmatlas.com/foldSequence/v1>). Similarity scores were then calculated using FoldSeek v6.29e2557 (van Kempen *et al.* 2024) against 141 PDB profiles identified as RdRp (listed in Supplementary Data).

PalmScan v1.0.i86linux64 (Babaian and Edgar, 2022) was used to search for core RdRp motifs in all proteins, with RdRp and RT detection enabled and otherwise the default settings.

Phylogenetic tree distances were calculated with the ete3 get_distance function as above. HMMER and BLAST scores were calculated as discussed above.

**Mito-like Virus** **Phylogeny**

Given that many EVEs are degraded and partial sequences are common, to ensure the accuracy of the phylogeny and avoid sequences clustering by which region of RdRp is present, only sequences fully overlapping the same 30 amino acid region and with a length of at least 100 amino acids after trimming were selected for phylogenetic analysis. A reliable alignment could not be generated based on only the newly identified sequences, so based on the BLAST analysis described in the main Materials and Methods section, the most similar known RdRp to each putative mito-like RdRp was identified and these known proteins were aligned using the MAFFT linsi algorithm (v7.520) (Katoh et al., 2002). The position of the uncharacterised protein BLAST hit on its target sequence was then used to establish the position of the uncharacterised protein on the mitoviral RdRp. The 30 amino acid region spanned by the greatest number of uncharacterised proteins was identified and the uncharacterised proteins fully overlapping this region selected for phylogenetic analysis.

This left 328 plant sequences. These sequences represent most plant orders in the original dataset, however EVEs from some, most notably the Asparagales, were too degraded to include. An additional 5 sequences from non-plant hosts overlapping this region were also included.

An alignment was created incorporating these uncharacterised proteins, *Mitoviridae* reference sequences and mito-like viruses identified in other virus discovery projects. Sequences were aligned using the default MAFFT algorithm. The overall *Mitoviridae* and mito-like virus phylogeny was built using FastTree2, with the default settings. Sequences were assigned to clades based on this phylogeny. Subtrees were extracted from the newick file of this phylogeny. The full mito-like virus phylogeny shown in Figure S2 is provided in newick format in the Supplementary Data.

Tanglegrams were calculated using Dendroscope (v3.8.10) (Huson *et al.* 2007) and visualised using plot_phylo (v0.0.4, [github.com/KatyBrown/plot_phylo](http://github.com/KatyBrown/plot_phylo)). Online Hhpred (Söding *et al.* 2005, Zimmermann *et al.* 2018) searches were performed using the server at <https://toolkit.tuebingen.mpg.de/tools/hhpred> against Pfam-A_v37 between 2024-04-01 and 2024-05-31.

**Mitochondrial Genome Comparisons**

To find the mitochondrial genome of species with specific endogenous mito-like elements, the NCBI genome database was searched on 2024-12-06 for the taxonomic labels “rosids” (taxonomy ID 71275), Zingiberales (4618) and Solanales (4069). Genomes were filtered to keep only reference genomes assembled to complete or chromosome level and only those with “Mitochondrion” in the organelle_info field of their NCBI genome record. Mitochondrial sequences were isolated as those with labels containing the string “mitochondrion” and complete sequences as those containing the string “complete”. One mitochondrial sequence was selected per genome, where there was more than one sequence labelled as mitochondrial, those labelled complete were prioritised, if none were complete the longest was selected. Each mitochondrial sequence was compared to the sequences shown in Figures 2E, 2G and 2H using TBLASTN via standalone BLAST and filtered to keep only hits with a bit score > 100, percent identity > 90 and alignment length > 100 nt, as sequences within this clade are very closely related. BLAST match regions separated by < 1000 nt were merged using BedTools (v2.31.1) (Quinlan and Hall, 2010). Every possible pairwise comparison was made between mitochondrial sequences containing putative EVEs and each pair was aligned using NUCMER (v3.1) (Kurtz *et al.* 2004) with relaxed settings (-l 15 -c 50 -g 200 -d 10 --maxmatch). Regions longer than 8,000 nt which were successfully aligned and contained EVEs were isolated.

For the mitoviral sequences shown in Figure S4, mito-like regions identified above using TBLASTN were extended by 5,000 nt in each direction using bedtools slop (Quinlan and Hall, 2010). ORFs longer than 100 amino acids were identified using orfipy (Singh and Wurtele, 2021). ORFs were identified using the HHpred online server (Söding *et al.* 2005, Zimmermann *et al.* 2018) (accessed 2025-04-07). Only the most significant HHpred result was selected for each ORF and only results with a HHpred score >50 were included.

**Orbivirus-Like Sequences**

Initial TBLASTX searches were performed using standalone BLAST with the protein sequences identified amongst uncharacterised proteins as queries and nematode reference genomes (listed in Supplementary Data) as targets. Sequences were aligned using the MAFFT linsi algorithm and sequence similarity matrices generated with CIAlign. Chromosome-to-chromosome alignments were generated using NUCMER (v3.1) (Kurtz *et al.* 2004) with a maximum gap size of 500 and a minimum cluster size of 100. Corresponding regions were isolated using BedTools (v2.31.1) (Quinlan and Hall, 2010). Visualisations of sequence alignments were generated using CIAlign.

RNA-seq datasets from nematodes containing potential reoviral transcripts were identified by querying the online Serratus database (accessed 2024-07-23, <https://serratus.io/>) (Edgar *et al.,* 2022). All datasets were downloaded from SRA (<https://www.ncbi.nlm.nih.gov/sra>) between 2024-08-07 and 2024-08-14. Reads were trimmed with trim_galore (v0.6.4_dev, <https://github.com/FelixKrueger/TrimGalore>) then mapped sequentially to various databases to remove non-viral reads as follows: invertebrate ribosomal RNA from the SILVA database (downloaded 2020-01-15), the appropriate host genome, the human reference genome (GRCh38.p7 primary assembly), and bacterial RefSeq genomes from NCBI (downloaded 2017-10-02). Mapping was performed with bowtie2 (v2.4.1) (Langmead and Salzberg, 2012), or hisat2 (for host and human) (v2.2.1) (Kim *et al.* 2015) both with the setting --ignore-quals and allowing 5% of the read length as mismatches. The unmapped reads after these filtering steps were assembled into contigs using the SPAdes genome assembler in rnaSPAdes mode (v3.15.5) (Bushmanova *et al.* 2019). ORFs were identified in the assembled contigs using the EMBOSS getorf function (v6.5.7.0) (Rice *et al.,* 2000).

A set of related orbi-like proteins to use to identify further nematode orbi-like viruses was identified initially by using BLASTP against the nr protein database on the NCBI online server (accessed 2024-07-25), using the initially identified *Brugia* uncharacterised proteins as queries. BLASTP results were not filtered as HHsearch was later applied to the results. The taxonomy IDs for these sequences were identified and, for each taxon, all NCBI sequences were downloaded. All NCBI RefSeq sequences for ICTV classified members of the *Orbivirus* genus were also included. The Pfam profiles for *Orbivirus* proteins (listed in the Supplementary Data) were used as targets for HHsearch (v3.3.0) (Steinegger *et al.* 2019), with the default settings, to classify which viral proteins these sequences correspond to. The proteins, their host species and their matching orbi-like virus profiles are listed in the Supplementary Data.

To identify RdRp in the assembled SRA contigs, they were compared to VP1 proteins from this set using DIAMOND BLASTP (v0.9.14) (Buchfink *et al.* 2021), with the default settings. For *H. contortus*, which has intact ORFs, the highest scoring RdRps from these results were used for all further analysis. For other nematodes, the identified contigs were used as input to miniprot below to reconstruct ORFs.

Miniprot (v0.13-r248) (Li, 2023) was used to reconstruct putative ORFs from degraded EVE sequences with relaxed settings as follows (as the sequences are expected to differ substantially from the reference) – no splicing (-S), kmer size (-k) and kmer size for second round of chaining (-l) 6, modimisers bit (seed density, -M) 0, frameshift penalty (-F) 6, minimum number of syncmers (-n) 6, minimum proportion of query aligned (--outc) 0.1. Chromosome regions identified above and the non-*H. contortus* SRA contigs were used as queries and the known *Orbivirus* and orbi-like virus VP1 proteins described above were initially used as target sequences. The process was repeated iteratively three times, with the ORFs resulting from the previous round as queries, to identify additional or longer regions. Only regions with at least 100 matching nucleotides were retained. Reconstructed ORFs are available in the Supplementary Data, as is the information required to regenerate these ORFs from the contigs. For SRA contigs, the contig sequences which contained the ORFs are also available in the Supplementary Data.

To identify additional segments in *H. contortus*, HMMscan and HHpred were used as described above for all *H. contortus* ORFs against the orbi-like virus Pfam profiles, plus against additional profiles generated by clustering (using MMSeqs with 35% similarity and 20% coverage in cluster mode 0) the unidentified proteins from known orbi-like viruses. Proteins scoring >30 with either of these approaches were then checked manually with online BLAST and compared using ESMfold and FoldSeek. For ESMfold, proteins were split into substrings with a length of 400 amino acids and an overlap of 200 amino acids. These sequences were folded using the ESM Fold Sequence online server (accessed via API on 2024-09-10 at <https://api.esmatlas.com/foldSequence/v1> (Lin et al. 2023). Similarity scores were then calculated using FoldSeek (v6.29e2557) (van Kempen *et al.* 2024) against structures generated using ESMfold, with the same settings, for the known orbi-like proteins. Sequences with a FoldSeek bit score >50 were additionally verified using the HHpred online server against Pfam-A_v37 (accessed 2024-09-13).

**CDD Annotations**

CDD (Yang *et al.* 2020) annotations for uncharacterised NCBI proteins were retrieved on 2024-09-16 and assigned as: RdRp – domain only associated with RNA viral RdRp, RT-like – domain associated with reverse transcriptase or a related annotation, RNA viral non-RdRp – domain associated only with RNA viral sequences but not with RdRp, RT-like – domain associated with reverse transcriptase or a related protein and other – not specifically RNA viral. Assignment of the annotations was via examining the taxonomy of the sequences in which the CDD domain has previously been identified and then manually checking which annotations fell into which group and comparing these to the online descriptions of the domains. Assignments are provided in Supplementary Table 1.

**Near Misses**

Near misses were defined as sequences which had a HMMER (Eddy, 2011) hit against a viral RdRp with a score score ≥10 and <25. There were 1,349,599 such sequences. These sequences were compared to the nr database using DIAMOND BLASTP and filtered to keep sequences with an alignment length of >100 amino acids and an identity >40% against any sequence not labelled as uncharacterised or similar (excluding self hits), these thresholds were deliberately relaxed as the exact nature of these sequences is not particularly relevant, only that they are unlikely to be viral. This left 577,704 sequences. A negative control dataset of similar size was generated for comparative purposes. To select the negative control sequences, a random sample of 1,000,000 unclassified sequences from the NCBI protein database was taken using the Unix shuf command, and these sequences were subjected to BLAST against the NCBI nr protein database with the same conditions as the near miss sequences, leaving 499,240 sequences. The BLAST target with the highest bit score against each query in both sets was selected.

To classify the BLAST target names into clusters, the full protein names were split into terms of two or more words, using a single space character as a delimiter. Where multiple overlapping terms were present in the same set of proteins, only the longest term was included. Terms appearing in 20 or more proteins in the near miss set were then clustered by overlap, and clusters were checked and edited manually to make biological sense, with synonyms and closely related terms grouped. Ambiguous terms were excluded. Clusters of terms and excluded terms are listed in the Supplementary Data. The number of proteins with names including any of the terms in each cluster was then counted, in the near miss and the negative control datasets. Clusters were considered to be significantly enriched if the p-value of a Fisher Exact Test (as implemented in scipy v1.10.1, Virtanen *et al.* 2020), multiplied by the number of clusters (62) to correct for multiple testing, was less than 0.05. To calculate fold changes, a small constant of 10^-5^ was added to the percentage of proteins annotated with the term, to account for zero counts in the denominator.

For the top ten annotations identified as enriched amongst near-miss proteins compared to the controls, BLAST target sequences matching near-miss proteins were compared directly to the relevant HMM profiles using HMMscan. The matching regions identified by HMMscan were extracted from both the profile alignments and the target sequence alignments. New HMMs were generated using just these regions of the profile alignments using HMMbuild and the target sequence regions were realigned to these HMMs using HMMalign with the --mapali setting. Sequence logos were generated using CIAlign.

**Figures**

Unless otherwise specified, subtrees in figures were generated by manually selecting clades from these large phylogenies, realigning sequences at higher stringency using the MAFFT linsi algorithm (v7.520) (Katoh *et al.* 2002), cropping with CIAlign (Tumescheit *et al.* 2022) to remove terminal regions with coverage <50% or similarity <30% and to remove sequences <50 amino acids in length, and rebuilding trees using FastTree as above, with additional reference sequences added by BLASTP searches against nr on 20/03/2024 and from the VMR_MSL39_v2 version of the ICTV metadata resource. Subtrees were visualised using plot_phylo (v0.0.4, [github.com/KatyBrown/plot_phylo](http://github.com/KatyBrown/plot_phylo)).

Altschul, S. F., Gish, W., Miller, W., Myers, E. W., & Lipman, D. J. (1990). Basic local alignment search tool. *Journal of molecular biology*, *215*(3), 403–410. https://doi.org/10.1016/S0022-2836(05)80360-2

Babaian, A., & Edgar, R. (2022). Ribovirus classification by a polymerase barcode sequence. *PeerJ*, *10*, e14055. https://doi.org/10.7717/peerj.14055

Buchfink, B., Reuter, K., & Drost, H. G. (2021). Sensitive protein alignments at tree-of-life scale using DIAMOND. *Nature methods*, *18*(4), 366–368. https://doi.org/10.1038/s41592-021-01101-x

Bushmanova, E., Antipov, D., Lapidus, A., & Prjibelski, A. D. (2019). rnaSPAdes: a de novo transcriptome assembler and its application to RNA-Seq data. *GigaScience*, *8*(9), giz100. https://doi.org/10.1093/gigascience/giz100

Camacho, C., Coulouris, G., Avagyan, V., Ma, N., Papadopoulos, J., Bealer, K., & Madden, T. L. (2009). BLAST+: architecture and applications. *BMC bioinformatics*, *10*, 421. https://doi.org/10.1186/1471-2105-10-421

Charon, J., Buchmann, J. P., Sadiq, S., & Holmes, E. C. (2022). RdRp-scan: A bioinformatic resource to identify and annotate divergent RNA viruses in metagenomic sequence data. *Virus evolution*, *8*(2), veac082. https://doi.org/10.1093/ve/veac082

Eddy S. R. (2011). Accelerated Profile HMM Searches. *PLoS computational biology*, *7*(10), e1002195. https://doi.org/10.1371/journal.pcbi.1002195

Edgar, R. C., Taylor, B., Lin, V., Altman, T., Barbera, P., Meleshko, D., Lohr, D., Novakovsky, G., Buchfink, B., Al-Shayeb, B., Banfield, J. F., de la Peña, M., Korobeynikov, A., Chikhi, R., & Babaian, A. (2022). Petabase-scale sequence alignment catalyses viral discovery. *Nature*, *602*(7895), 142–147. https://doi.org/10.1038/s41586-021-04332-2

Huerta-Cepas, J., Serra, F., & Bork, P. (2016). ETE 3: Reconstruction, Analysis, and Visualization of Phylogenomic Data. *Molecular biology and evolution*, *33*(6), 1635–1638. https://doi.org/10.1093/molbev/msw046

Huson, D. H., Richter, D. C., Rausch, C., Dezulian, T., Franz, M., & Rupp, R. (2007). Dendroscope: An interactive viewer for large phylogenetic trees. *BMC bioinformatics*, *8*, 460. https://doi.org/10.1186/1471-2105-8-460

Kans J. Entrez Direct: E-utilities on the Unix Command Line. 2013 Apr 23 [Updated 2025 Mar 25]. In: Entrez Programming Utilities Help [Internet]. Bethesda (MD): National Center for Biotechnology Information (US); 2010-. Available from: <https://www.ncbi.nlm.nih.gov/books/NBK179288/>

Katoh, K., Misawa, K., Kuma, K., & Miyata, T. (2002). MAFFT: a novel method for rapid multiple sequence alignment based on fast Fourier transform. *Nucleic acids research*, *30*(14), 3059–3066. https://doi.org/10.1093/nar/gkf436

Kim, D., Langmead, B., & Salzberg, S. L. (2015). HISAT: a fast spliced aligner with low memory requirements. *Nature methods*, *12*(4), 357–360. https://doi.org/10.1038/nmeth.3317

Kurtz, S., Phillippy, A., Delcher, A. L., Smoot, M., Shumway, M., Antonescu, C., & Salzberg, S. L. (2004). Versatile and open software for comparing large genomes. *Genome biology*, *5*(2), R12. https://doi.org/10.1186/gb-2004-5-2-r12

Langmead, B., & Salzberg, S. L. (2012). Fast gapped-read alignment with Bowtie 2. *Nature methods*, *9*(4), 357–359. https://doi.org/10.1038/nmeth.1923

Li H. (2023). Protein-to-genome alignment with miniprot. *Bioinformatics*, *39*(1), btad014. https://doi.org/10.1093/bioinformatics/btad014

Lin, Z., Akin, H., Rao, R., Hie, B., Zhu, Z., Lu, W., Smetanin, N., Verkuil, R., Kabeli, O., Shmueli, Y., Dos Santos Costa, A., Fazel-Zarandi, M., Sercu, T., Candido, S., & Rives, A. (2023). Evolutionary-scale prediction of atomic-level protein structure with a language model. *Science*, *379*(6637), 1123–1130. https://doi.org/10.1126/science.ade2574

Obbard, D. J., Shi, M., Roberts, K. E., Longdon, B., & Dennis, A. B. (2020). A new lineage of segmented RNA viruses infecting animals. *Virus evolution*, *6*(1), vez061. https://doi.org/10.1093/ve/vez061

Olendraite, I., Brown, K., & Firth, A. E. (2023). Identification of RNA Virus-Derived RdRp Sequences in Publicly Available Transcriptomic Data Sets. *Molecular biology and evolution*, *40*(4), msad060. https://doi.org/10.1093/molbev/msad060

Price, M. N., Dehal, P. S., & Arkin, A. P. (2010). FastTree 2--approximately maximum-likelihood trees for large alignments. *PloS one*, *5*(3), e9490. https://doi.org/10.1371/journal.pone.0009490

Quinlan, A. R., & Hall, I. M. (2010). BEDTools: a flexible suite of utilities for comparing genomic features. *Bioinformatics*, *26*(6), 841–842. https://doi.org/10.1093/bioinformatics/btq033

Rice, P., Longden, I., & Bleasby, A. (2000). EMBOSS: the European Molecular Biology Open Software Suite. *Trends in genetics : TIG*, *16*(6), 276–277. https://doi.org/10.1016/s0168-9525(00)02024-2

Shi, M., Lin, X. D., Tian, J. H., Chen, L. J., Chen, X., Li, C. X., Qin, X. C., Li, J., Cao, J. P., Eden, J. S., Buchmann, J., Wang, W., Xu, J., Holmes, E. C., & Zhang, Y. Z. (2016). Redefining the invertebrate RNA virosphere. *Nature*, *540*(7634), 539–543. https://doi.org/10.1038/nature20167

Singh, U., & Wurtele, E. S. (2021). orfipy: a fast and flexible tool for extracting ORFs. *Bioinformatics*, *37*(18), 3019–3020. https://doi.org/10.1093/bioinformatics/btab090

Söding, J., Biegert, A., & Lupas, A. N. (2005). The HHpred interactive server for protein homology detection and structure prediction. *Nucleic acids research*, *33*(Web Server issue), W244–W248. https://doi.org/10.1093/nar/gki408

Steinegger, M., & Söding, J. (2017). MMseqs2 enables sensitive protein sequence searching for the analysis of massive data sets. *Nature biotechnology*, *35*(11), 1026–1028. https://doi.org/10.1038/nbt.3988

Steinegger, M., Meier, M., Mirdita, M., Vöhringer, H., Haunsberger, S. J., & Söding, J. (2019). HH-suite3 for fast remote homology detection and deep protein annotation. *BMC bioinformatics*, *20*(1), 473. https://doi.org/10.1186/s12859-019-3019-7

Tumescheit, C., Firth, A. E., & Brown, K. (2022). CIAlign: A highly customisable command line tool to clean, interpret and visualise multiple sequence alignments. *PeerJ*, *10*, e12983. https://doi.org/10.7717/peerj.12983

van Kempen, M., Kim, S. S., Tumescheit, C., Mirdita, M., Lee, J., Gilchrist, C. L. M., Söding, J., & Steinegger, M. (2024). Fast and accurate protein structure search with Foldseek. *Nature biotechnology*, *42*(2), 243–246. https://doi.org/10.1038/s41587-023-01773-0

Virtanen, P., Gommers, R., Oliphant, T. E., Haberland, M., Reddy, T., Cournapeau, D., Burovski, E., Peterson, P., Weckesser, W., Bright, J., van der Walt, S. J., Brett, M., Wilson, J., Millman, K. J., Mayorov, N., Nelson, A. R. J., Jones, E., Kern, R., Larson, E., Carey, C. J., … SciPy 1.0 Contributors (2020). SciPy 1.0: fundamental algorithms for scientific computing in Python. *Nature methods*, *17*(3), 261–272. https://doi.org/10.1038/s41592-019-0686-2

Yang, M., Derbyshire, M. K., Yamashita, R. A., & Marchler-Bauer, A. (2020). NCBI's Conserved Domain Database and Tools for Protein Domain Analysis. *Current protocols in bioinformatics*, *69*(1), e90. https://doi.org/10.1002/cpbi.90

Zimmermann, L., Stephens, A., Nam, S. Z., Rau, D., Kübler, J., Lozajic, M., Gabler, F., Söding, J., Lupas, A. N., & Alva, V. (2018). A Completely Reimplemented MPI Bioinformatics Toolkit with a New HHpred Server at its Core. *Journal of molecular biology*, *430*(15), 2237–2243. https://doi.org/10.1016/j.jmb.2017.12.007
